# Resonance-Anchored Residual Surrogate for Spectral Prediction and Inverse Design of Multiple Terahertz Metamaterial Absorber Topologies

**DOI:** 10.3390/nano16171107

**Published:** 2026-09-03

**Authors:** Guilin Li, Weiwei Qu, Yan Huang, Yurong Wang, Qin Yi, Hu Deng

**Affiliations:** School of Information and Control Engineering, Southwest University of Science and Technology, Mianyang 621010, China; liguilin@mails.swust.edu.cn (G.L.); quweiwei@swust.edu.cn (W.Q.); huangyan313@swust.edu.cn (Y.H.); wangyurong@mails.swust.edu.cn (Y.W.); yee1128@mails.swust.edu.cn (Q.Y.)

**Keywords:** terahertz metamaterial absorber, multi-topology modeling, resonance anchored, residual spectral prediction, PGD inverse design

## Abstract

Efficient forward prediction and inverse design can bypass the complexity and computational cost of traditional full-wave simulations, accelerating the development of terahertz metamaterial absorbers. Most current machine-learning approaches for spectral prediction or inverse design are limited to absorbers with a single topology, requiring retraining whenever the topology changes. Simultaneous forward prediction and inverse design also require separate models that are trained independently. This study proposes a resonance-anchored residual surrogate framework for resonance-frequency prediction and goal-oriented inverse design across multiple absorber topologies. Unlike conventional spectral prediction, the proposed spectral representation consists of a resonance frequency and a continuous local absorption spectrum centered on the resonance peak. The residual network is trained using a physics-guided dynamic anchor loss to improve the accuracy of resonance-frequency prediction, local spectral reconstruction, peak alignment, and absorptivity estimation. On the test set, the model achieves a normalized resonance-frequency MSE of 6.64 × 10^−5^, a normalized spectral-waveform MSE of 3.58 × 10^−3^, and a physical-domain absorptivity RMSE of 0.0054. Ablation experiments verify the effectiveness of the residual connections and the dynamic anchor loss. The trained surrogate is then frozen and integrated into a projected gradient descent loop for inverse design. Full-wave simulations demonstrate that the optimized structures successfully reproduce the target resonance responses within the sampled design space.

## 1. Introduction

Terahertz waves, which occupy the 0.1–10 THz range between microwaves and the infrared region, have attracted increasing interest due to their advantageous properties, including penetration through non-conductive materials, low photon energy, and fingerprint spectral recognition [1,2,3]. Harnessing these properties in practical devices requires flexible electromagnetic control, which is difficult to achieve with natural materials because their terahertz tunability is inherently limited. Metamaterials, through artificially designed subwavelength structures, overcome this limitation and deliver tailored electromagnetic responses. Because the characteristic dimensions of resonant metamaterials scale with their operating wavelength, terahertz meta-atoms naturally exhibit micrometer-scale lateral dimensions while remaining subwavelength relative to the incident wavelength. Owing to their simple structure and high absorption, terahertz metamaterial absorbers exploit this design flexibility to achieve strong field confinement, narrowband resonance, and high absorptivity [4,5,6], which underpin their wide applications in photovoltaics [7,8], thermal emitters [9], sensing [10], stealth technology [11], and surface-enhanced Raman spectroscopy [12].

The design of terahertz metamaterial absorbers principally follows a conventional workflow consisting of structural design, electromagnetic simulation, parameter optimization, and performance enhancement [13,14,15,16,17,18]. This workflow relies on full-wave electromagnetic simulations and iterative parameter optimization to achieve superior absorption performance. It is limited by low design efficiency and high computational cost. Heuristic optimization algorithms, such as particle swarm optimization [19], genetic algorithms [20,21], and firefly algorithms [22], have been introduced to reduce manual trial-and-error in metamaterial design. These methods still require repeated full-wave simulations to evaluate candidate structures during iterative optimization. The computational burden becomes more pronounced when the design involves large parameter spaces, multiple topology classes, or simultaneous constraints on resonance frequency, peak absorptivity, bandwidth, and quality factor. These requirements are also strongly application-dependent: high-Q sensing requires accurately positioned narrow resonances and strong field confinement, whereas stealth and radar-cross-section reduction emphasize high absorptivity together with bandwidth, angular, and polarization robustness; photovoltaic and selective-thermal-emission absorbers place greater emphasis on broadband or spectrally selective energy coupling and material-compatible design [7,8,9,10,11,12]. Consequently, efficient design methods are needed not only to reduce simulation cost but also to rapidly explore application-specific trade-offs among resonance position, bandwidth, absorptivity, robustness, and feasible geometry.

Recent advances in deep-learning surrogate modelling have extended beyond fixed-topology parameter-to-spectrum regression towards more flexible forward and inverse design strategies, including attention- and Transformer-based spectral prediction, generative free-form inverse design, transfer-learning-enabled reusable models, and surrogate-assisted optimization over discrete topology spaces [23,24,25,26,27,28]. These developments have broadened the range of learnable structural representations and optimization strategies for metamaterial design. By learning the nonlinear mapping between structural parameters and electromagnetic responses, surrogate models can significantly reduce the computational effort required for full-wave simulations and parameter sweeps [29,30,31,32,33,34,35,36]. Nevertheless, several challenges remain for narrowband terahertz absorbers with diverse structural topologies. First, many reported learning-based design methods are still developed for a fixed topology or a specific resonance pattern, and their predictive performance is generally established within the geometry, material, frequency, and objective distributions represented in the training data. When the resonator topology changes, separate topology-specific surrogate models or additional retraining may therefore be required, increasing model redundancy and complicating unified forward prediction and inverse design [37,38,39,40,41]. Second, inverse design is intrinsically non-unique because different geometry–topology combinations may produce similar target spectra [39,42,43,44]. Directly training an inverse network may therefore suffer from ambiguity caused by one-to-many mappings. Although generative models such as GANs [45] and VAEs [46] can provide multiple candidate designs, they also introduce issues such as training instability and limited interpretability of the latent space [47,48,49]. The generated structures still require additional validation to ensure physical feasibility and consistency with the target response [50,51]. Another important issue lies in the spectral representation and loss formulation. Many spectral prediction models directly regress the full absorption spectrum using pointwise losses. For narrowband absorbers, broad off-resonance regions may dominate the full-spectrum mean squared error, while the physically important resonance peak position and peak absorptivity remain insufficiently constrained. A small horizontal shift in the resonance peak can lead to a noticeable mismatch between the designed and target operating bands, even when the averaged spectral error is low. A resonance-aware spectral representation and a physics-guided loss function are needed to improve resonance-level prediction accuracy.

To address these issues, this work proposes a resonance-anchored residual surrogate framework for the forward prediction and inverse design of multiple predefined terahertz metamaterial absorber topology classes. First, a resonance-anchored spectral representation is introduced to describe each absorption response using the dominant resonance frequency and a local absorption profile centered on the resonance peak. Second, a residual fully connected surrogate model is trained using a physics-guided dynamic anchor loss to jointly constrain the resonance frequency, local spectral shape, peak-center alignment, peak absorptivity, and absorptivity bounds. Third, instead of training a separate inverse network, the validated forward surrogate is frozen and used as a differentiable design engine. Projected gradient descent is then applied to optimize only the continuous geometric parameters, while different topology labels are searched independently. The proposed framework is evaluated through blind-test prediction, topology-wise reconstruction, ablation analysis, physical consistency analysis, and full-wave simulation verification of inverse-designed candidates. This design method combines high design efficiency, accurate absorption-spectrum prediction, and physical interpretability, providing a viable approach for unified topology-conditioned modeling and inverse design across multiple supported terahertz metamaterial absorber classes.

## 2. Materials and Methods

### 2.1. Metamaterial Absorber Structure and Data Set Acquisition

**Figure 1:**
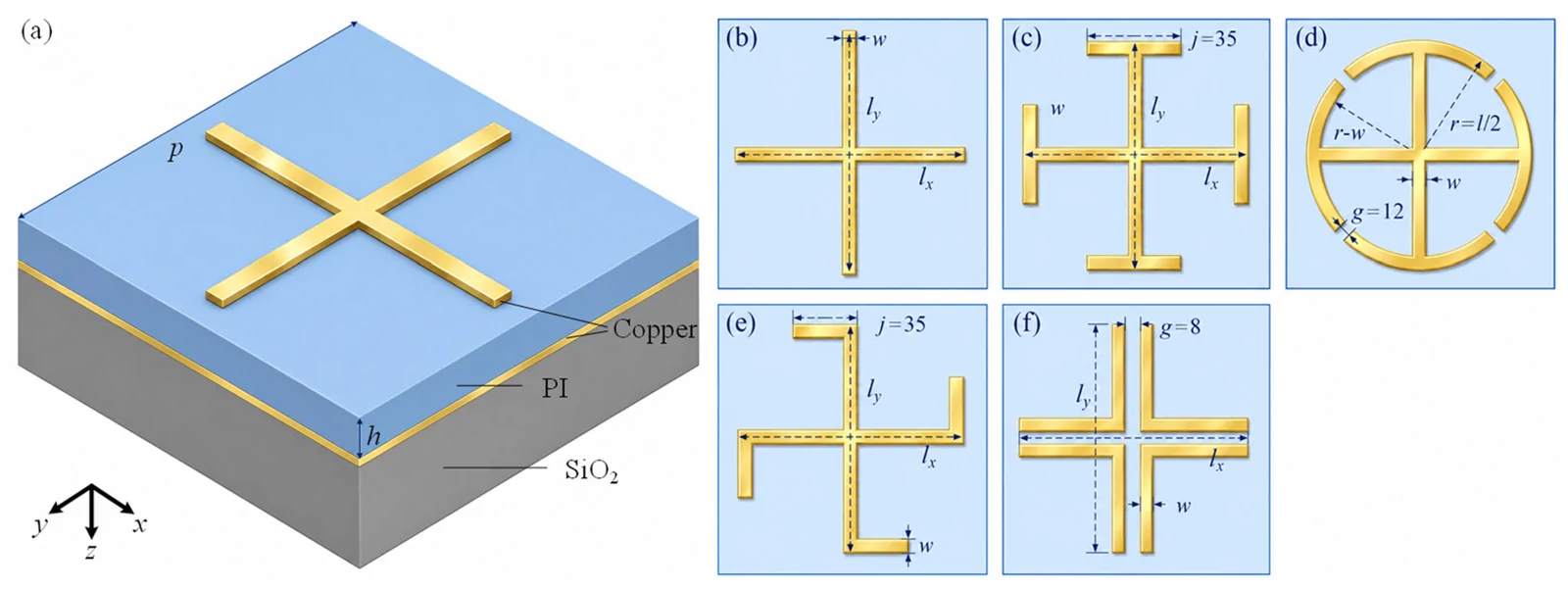
Schematic diagram of absorber structure. (**a**) Three-dimensional view of the absorber structure. (**b**) Standard Cross. (**c**) Jerusalem Cross. (**d**) Cross-Enclosed Split Ring. (**e**) Chiral Cross (**f**) Split-Cross.

To verify the proposed residual-network-based surrogate-assisted design framework, this study comprehensively evaluates its forward prediction and inverse design capabilities using a terahertz metamaterial absorber. A typical terahertz metamaterial absorber structure is shown in Figure 1a. The upper metal resonant layer and the lower metal reflective layer of the metamaterial absorber act as two parallel Bragg reflectors, while the dielectric layer between them forms a Fabry–Pérot cavity. Both the resonant pattern and the reflective layer are made of copper with a conductivity (σ) of 5.96 × 10^7^ S/m. Polyimide (PI), with a relative dielectric constant (ε) of 3.5 and a loss tangent (tan δ) of 0.0027, serves as the intermediate dielectric layer. The study showed that the metal layer thickness had no significant effect on the absorption characteristics. According to the skin depth formula, the metal layer thickness was set to 0.2 μm. Silicon dioxide serves as the substrate for the metamaterial absorber.

The electromagnetic behavior of metamaterial absorbers is fundamentally governed by their structural topology and material properties. Even absorbers made from the same materials and with similar resonant patterns can exhibit notable differences in absorption characteristics, including variations in resonance frequency and peak absorptivity. The resonant patterns on the top layer of the metamaterial absorber consist of five structures—Standard Cross, Jerusalem Cross, Cross-Enclosed Split Ring, Chiral Cross, and Split-Cross—as illustrated in Figure 1b–f. These five resonant structures are obtained through nesting, rotation, and division of the Standard Cross structure. To facilitate network training and dataset construction, category labels are assigned to the five resonant pattern structures: Standard Cross is labeled “1,” Jerusalem Cross is labeled “2,” Cross-Enclosed Split Ring is labeled “3,” Chiral Cross is labeled “4,” and Split-Cross is labeled “5.” To address the scale mismatch between the neural network input and discrete topological structures, one-hot encoding is employed to transform the labels into numerical feature vectors, such as representing “Standard Cross” as [1, 0, 0, 0, 0]. The resonant structure of a terahertz metamaterial absorber is characterized by four parameters: period (*p*), length of the metallic resonant pattern (*l*), line width (*w*), and thickness of the dielectric layer (*h*).

Training neural networks depends on data, making the acquisition of absorption spectra for different structural parameters essential. Full-wave simulations were performed using Computer Simulation Technology (v2021), with periodic boundary conditions applied along the *x*- and *y*-axes and open boundary conditions applied along the *z*-axis. The absorption coefficient can be determined using the following formula:

(1) $$A(\omega )=1-R(\omega )-T(\omega )=1-{\left|S_{11}\right|}^{2}-{\left|S_{21}\right|}^{2}$$

where *R*(*ω*) denotes the reflectance, *T*(*ω*) denotes the transmittance, and ω represents the frequency of the incident terahertz wave. *S*_11_ and *S*_21_ represent the reflection and transmission coefficients, respectively. Due to the metallic reflective layer, the transmittance approaches zero. The absorption coefficient can therefore be expressed as follows:

(2) $$A(\omega )=1-R(\omega )=1-{\left|S_{11}\right|}^{2}$$

Modeling in Computer Simulation Technology is often laborious and time-consuming for large-scale simulations. To streamline this process, joint simulations using Computer Simulation Technology (v2021) and MATLAB (v2025) were conducted to obtain the spectra required for training. The sampling step size was 2 for *p* and *l* and 1 for the remaining parameters. The sampling ranges for each structure are listed in Table 1. Each structure was sampled at 2000 design points (10 × 10 × 5 × 4), resulting in a total of 10,000 datasets across the five structures.

### 2.2. Resonance-Anchored Spectral Representation

**Figure 2:**
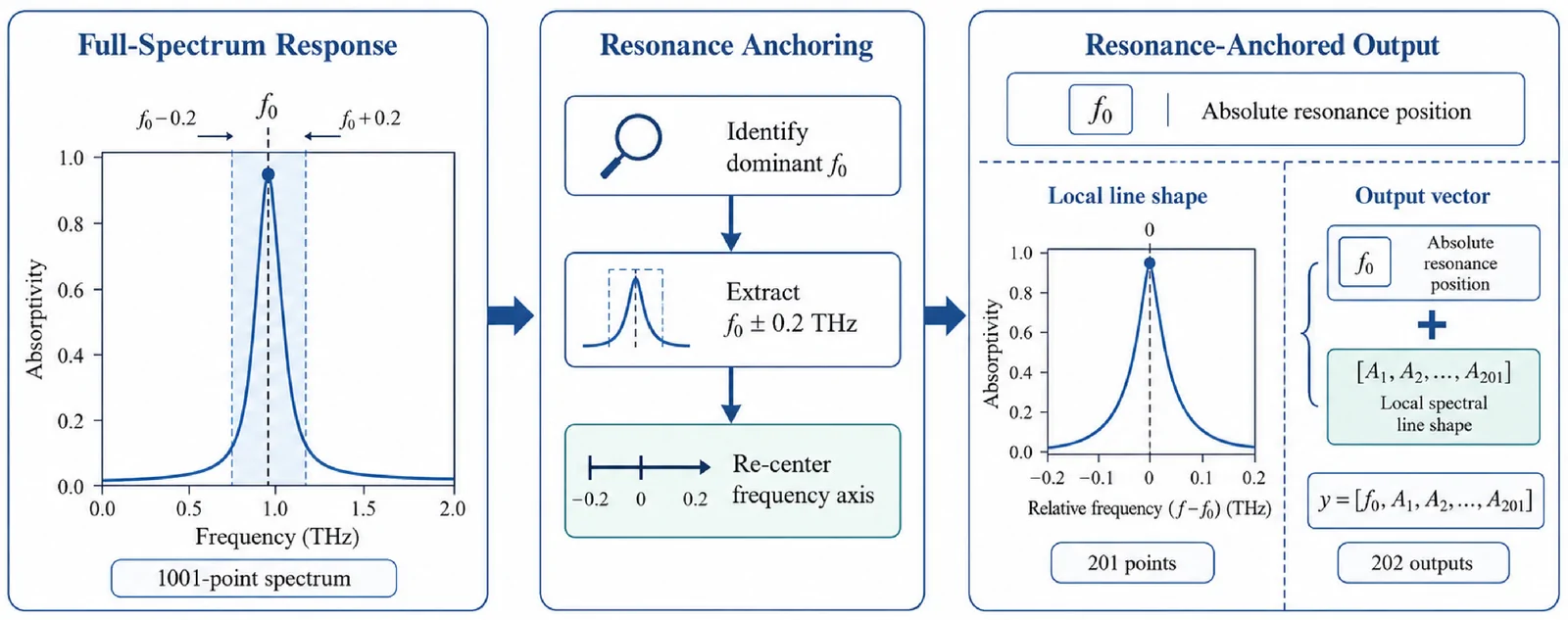
Schematic of the resonance-anchored spectral representation. The dominant resonance frequency *f*_0_ is separated from the local spectral line shape, and the 0.4 THz window centered at *f*_0_ is re-expressed using relative frequency coordinates.

Direct full-spectrum prediction represents the spectral response on a fixed global frequency grid, where extensive off-resonance regions contribute to the pointwise regression error despite containing comparatively limited resonance information. To provide a more resonance-oriented learning target, we introduce the resonance-anchored spectral representation illustrated in Figure 2.

For each simulated spectrum, the dominant resonance frequency *f*_0_ is first identified and retained as an independent scalar output. A 0.4 THz local window centered at *f*_0_, i.e., [*f*_0_ − 0.2, *f*_0_ + 0.2] THz, is then extracted from the original spectrum. With a frequency interval of Δ*f* = 0.002 THz, this window contains 201 spectral points. After re-centering the local frequency coordinate relative to *f*_0_, the surrogate output is expressed as *y* = [*f*_0_, *A*_1_, *A*_2_, …, *A*_201_], where the physical frequency of the *i*-th local point is reconstructed as *f_i_* = *f*_0_ + (*i* − 101)Δ*f*, *i =* 1, …, 201.

Unlike conventional fixed-grid full-spectrum prediction, this formulation explicitly separates the absolute resonance position from the local spectral line shape. Therefore, the proposed method changes the spectral representation itself rather than merely assigning greater loss weight to resonance-related frequency points.

### 2.3. Workflow of Forward Prediction and Frozen-Surrogate Inverse Design

**Figure 3:**
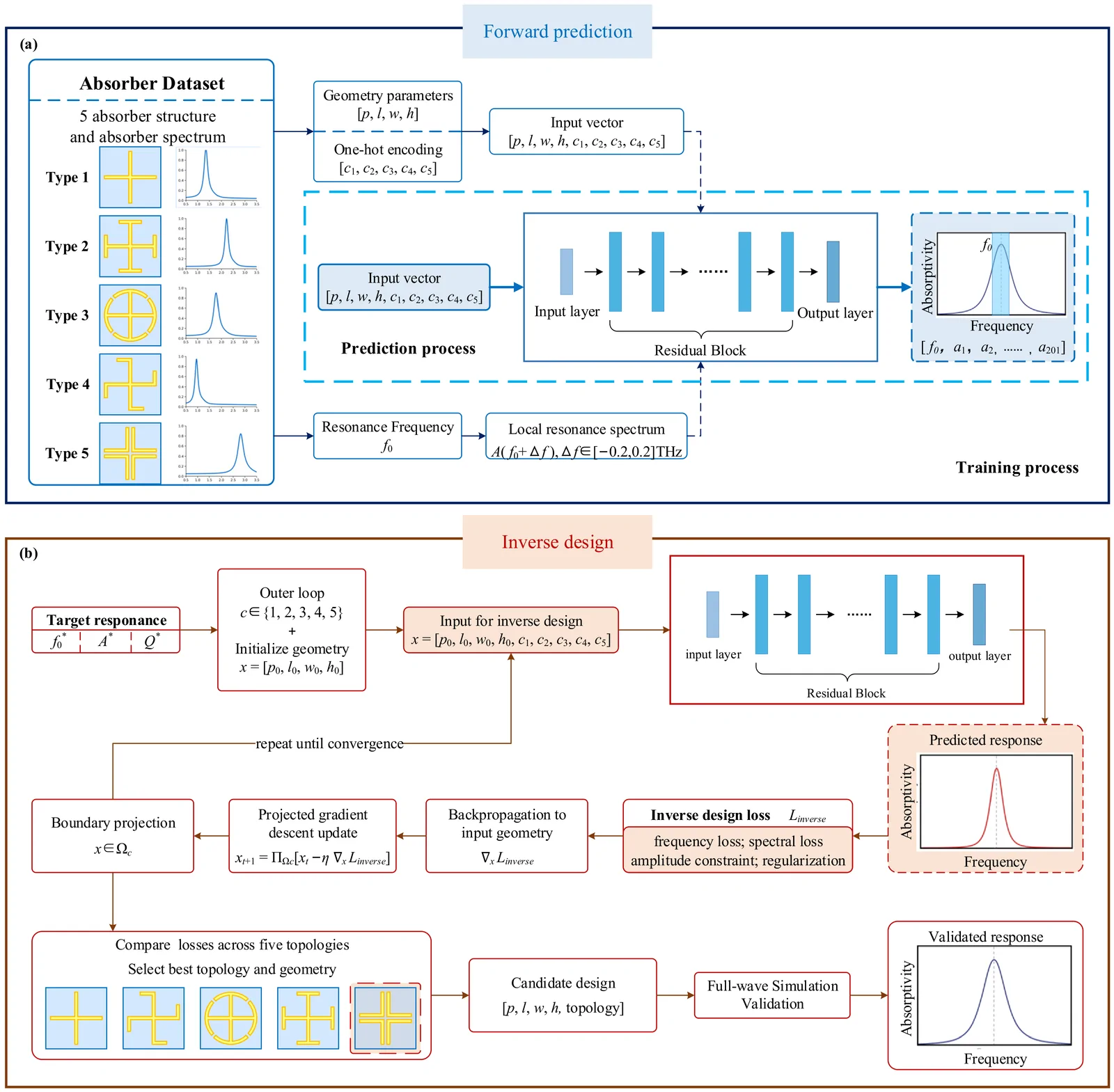
Workflow of the resonance-anchored surrogate-assisted design framework. (**a**) Full-wave-simulated absorber spectra are converted into resonance-anchored representations consisting of a single center frequency and a 201-point local absorption profile. Together with the geometric parameters and one-hot topology labels, these representations are used to train the residual forward surrogate network to predict the center frequency and local spectrum. (**b**) During inverse design, the pretrained forward surrogate network is frozen and used as a differentiable surrogate. For each topology class, projected gradient descent updates only the continuous geometric parameters to minimize the inverse-design loss. After convergence, the optimized losses are compared across the five topologies, the best design is selected, and full-wave simulation verification is performed.

Figure 3 illustrates the forward prediction and inverse design processes for terahertz metamaterial absorbers. First, a resonance-anchored forward surrogate model is trained, as shown in Figure 3a. The trained forward model is then frozen and repurposed as a differentiable function for projected-gradient-descent-based inverse design, as shown in Figure 3b. The forward prediction network maps the nine-dimensional input vector, consisting of four geometric parameters and a five-dimensional topology code, *x* = [*p*, *l*, *w*, *h*, *c*_1_, *c*_2_, *c*_3_, *c*_4_, *c*_5_], to the 202-dimensional resonance-anchored spectral output, *y* = [*f*_0_, *A*_1_, *A*_2_, …, *A*_201_]. To improve spectral prediction accuracy, the predicted spectrum is reconstructed on the physical frequency axis using the resonance-anchored representation. During inverse design, the target response is specified by the target resonance frequency, absorptivity, and quality factor *Q*. The pretrained forward surrogate network is kept frozen, and only the continuous geometric variables are updated. Because the topology label is discrete, the five absorber topologies are optimized independently by fixing their one-hot labels, after which the final candidates are compared according to the inverse-design loss. Since the reliability of the inverse-design stage depends on the forward surrogate, the architecture, loss formulation, and training protocol of the forward model are described first, followed by the inverse-design strategy.

The forward surrogate is a residual fully connected network inspired by residual learning. The model maps the nine-dimensional input to a 512-dimensional latent space, passes it through eight residual blocks, and outputs the resonance frequency and local spectrum. Residual connections can effectively model the strong nonlinear mapping from low-dimensional geometric variables to high-dimensional spectral responses. Compared with a standard multilayer perceptron, a residual network enables each block to learn a gradual correction to the underlying representation, improving gradient propagation and stabilizing the optimization of deeper fully connected networks. This capability is particularly useful for learning variations in cross-topology electromagnetic responses. Detailed layer settings are provided in Supplementary Section S1.

The loss function combines five terms that constrain resonance-frequency error, local spectral waveform error, peak-center alignment, peak-amplitude error, and absorptivity-bound violations. This design is motivated by the fact that narrowband absorbers are sensitive not only to pointwise waveform errors but also to small resonance shifts and peak-amplitude deviations. The complete mathematical definition, loss weights, and differentiable soft-argmax implementation are provided in Supplementary Section S1.

The complete dataset was divided into training, validation, and independent test sets using a stratified 8:1:1 split. All continuous inputs and outputs were standardized using statistics computed only from the training set. The model was trained using the AdamW optimizer with a learning rate of 0.0001 and a batch size of 128. To improve convergence and prevent overfitting, a cosine-annealing warm-restart scheduler and an early-stopping strategy were employed. During training, forward prediction accuracy was evaluated using the normalized mean squared error (MSE) for the resonance frequency and spectral waveforms. After inverse normalization, the evaluation included physically meaningful metrics such as the root mean squared error (RMSE) of the resonance frequency, R^2^, spectral RMSE, spectral waveform R^2^, and maximum peak-amplitude error.

To quantify the statistical uncertainty of the reported test-set performance, 95% confidence intervals were estimated using 5000 stratified nonparametric bootstrap resamples of the independent test set. Each complete absorber sample, including its resonance frequency and 201-point local spectrum, was treated as a single resampling unit, while the topology-wise sample proportions were preserved. The 2.5th and 97.5th percentiles of the bootstrap distributions were used as the confidence bounds.

To separately assess the reproducibility of the surrogate with respect to random data partitioning and network initialization, ten additional independent training runs were performed. For each run, the topology-stratified 8:1:1 train–validation–test split was regenerated, and the network was trained from a new random initialization, while the model architecture and training settings were kept unchanged. The complete repeated-training protocol and statistical results are provided in Supplementary Section S3.2.

After the forward surrogate model is trained and validated, it is frozen and integrated into a gradient-based inverse-design loop. Unlike inverse neural networks, which directly map spectra to structures, this method treats the continuous geometric parameters as optimization variables, updating them through backpropagation while keeping the network weights fixed. The inverse design treats *x* = [*p*, *l*, *w*, *h*] as the continuous variables to be optimized, with the topology label fixed during each projected gradient descent (PGD) cycle. The objective function penalizes deviations from the target resonance frequency, peak absorptivity, and quality factor (*Q*), while enforcing topology-specific boundary conditions. After each gradient step, the design variables are projected back into their feasible parameter ranges. To reduce the dependence of the local gradient-based optimization on a single initial geometry, PGD is performed from 10 initial geometries for each topology, including one topology-specific mean initialization and nine randomly sampled initializations within the feasible design bounds. Detailed settings are provided in Supplementary Section S2 and Table S3.

After convergence, each topology yields a candidate geometry. The candidates are ranked according to the final inverse-design loss, and the best-performing topology–geometry combination is selected for verification. When multiple solutions have comparable losses, the top-k candidates can be retained to reflect the one-to-many nature of the inverse problem. The optimized candidate is reconstructed for full-wave verification. The full-wave simulation spectrum is compared with both the target response and the surrogate-predicted response using the resonance-frequency deviation, peak-amplitude error, and local spectral RMSE. Because the frozen surrogate is validated primarily within the topology-specific geometric ranges represented in the training dataset, this projection also constrains the inverse-design search to the data-supported design domain and avoids relying on unvalidated surrogate behavior far outside the sampled parameter space.

## 3. Results and Discussion

### 3.1. Forward Prediction Accuracy on the Test Set

To comprehensively evaluate the forward prediction capability of the proposed physics-constrained residual surrogate model, validation was conducted using an independent test set. The training and validation losses decreased smoothly and showed no obvious signs of overfitting. The convergence curves are provided in Supplementary Figure S2.

On the test set, the proposed model achieved a normalized MSE of 6.64 × 10^−5^ (95% CI: 5.71 × 10^−5^–7.63 × 10^−5^) for the resonance center frequency and 3.58 × 10^−3^ (95% CI: 3.38 × 10^−3^–3.78 × 10^−3^) for the 201-point local spectral waveform. After inverse normalization, the mean local-window absorptivity RMSE was 0.0054 (95% CI: 0.0052–0.0055). These results indicate that the model can accurately predict both the resonance center frequency and the local absorption response around the resonance peak. Because the predicted center frequency is used to reconstruct the physical frequency axis of the local spectrum, accurate center-frequency prediction is essential for reliable spectral reconstruction.

The additional reproducibility assessment across ten independent training runs showed consistent predictive performance. The resonance-frequency RMSE was 0.0028 ± 0.0005 THz, and the mean local spectral RMSE was 0.0053 ± 0.0002. These values are consistent with the performance of the primary model, indicating that the reported prediction accuracy is not dependent on a particular random data partition or network initialization. Detailed run-wise results are provided in Supplementary Section S3.2 and Table S4.

**Figure 4:**
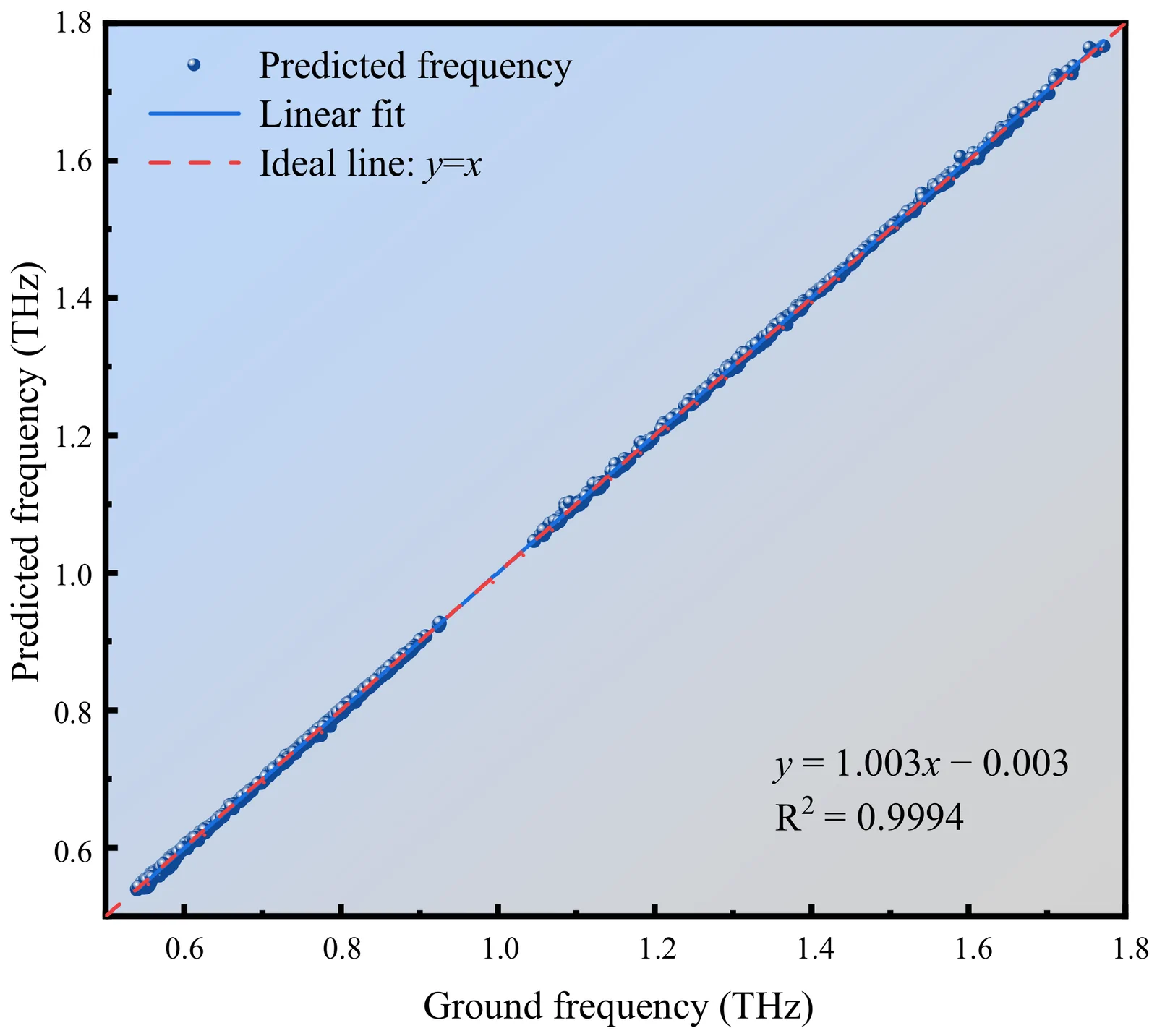
Regression analysis of the predicted and simulated resonance frequencies on the test set.

The regression performance for the resonance center frequency is further summarized in Figure 4. The predicted values closely align with the ideal diagonal, indicating strong agreement between the model predictions and the simulation results over the tested frequency range. The small deviation from the diagonal confirms that the resonance-anchored representation effectively separates the global resonance shift from the local spectral shape. This characteristic is important for terahertz absorber design because even a small resonance-frequency shift can lead to a noticeable mismatch between the designed and target operating bands.

Overall, the test results demonstrate that the trained forward surrogate provides a high-fidelity approximation of the full-wave-simulated electromagnetic response within the sampled design space. These results should be interpreted within the parameter ranges and topology classes included in the training dataset. Extrapolation to unseen geometries, new resonator topologies, or different material systems requires additional validation or extension of the dataset. Accordingly, the currently trained surrogate is intended primarily for high-fidelity prediction within the predefined, data-supported topology and geometric domains. The demonstrated advantage of the present framework lies in unified resonance-oriented modeling and surrogate-assisted inverse design across multiple predefined topology classes, rather than in unrestricted extrapolation beyond the data-supported design domain.

The proposed framework is not inherently limited to the present four geometric variables or five predefined topology classes. Higher-dimensional geometric parameters and additional material properties can be incorporated by expanding the input vector, although maintaining comparable prediction accuracy would require sufficient training-data coverage of the enlarged design space. Additional parametrically defined topologies can likewise be included by extending the topology encoding and geometric parameterization. For highly free-form or pixelated structures, richer structural representations, such as image-, mask-, graph-, or latent-space-based encodings, would be required. Therefore, the scalability of the framework depends not only on the network architecture but also on adequate data coverage and an appropriate representation of the expanded design space.

### 3.2. Spectral Reconstruction and Topology-Structure Generalization

In addition to predicting resonance frequencies, accurately reconstructing local absorption waveforms is essential for assessing whether the predictive model preserves the physically meaningful spectral line shapes of the absorber response. In the proposed resonance-anchored representation, each spectrum is described by a predicted resonance frequency and 201 absorptivity values within a local frequency window centered on that frequency. The accuracy of spectral reconstruction is therefore evaluated not only by the point-by-point waveform error but also by the consistency of the peak amplitude, peak width, and spectral profile.

**Figure 5:**
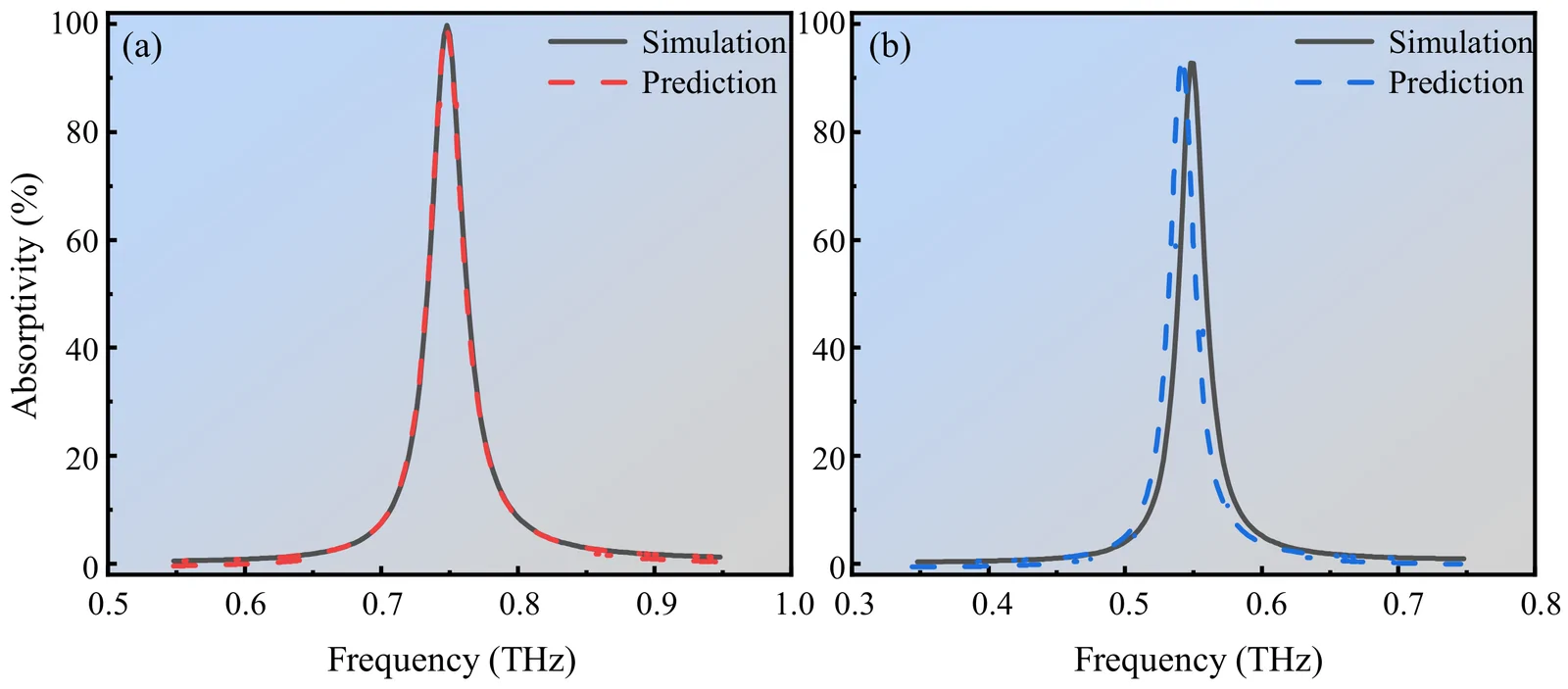
Best and worst spectral reconstruction cases on the test set. (**a**) Best prediction; (**b**) Worst prediction.

Figure 5 compares the simulated and predicted absorption spectra for the best (Figure 5a) and worst (Figure 5b) reconstruction cases in the test set. In the best case, the predicted curve closely matches the simulated spectrum, and the resonance peak position, peak absorptivity, and spectral shape are accurately captured. In the worst case, although the resonance peak position differs by 0.006 THz, the peak profile still agrees well with the simulation. This comparison demonstrates that the proposed model effectively predicts the resonance frequency and accurately reconstructs the local absorption waveform.

The topology-wise prediction results are summarized in Table 2 Each topology contributes 200 test samples, enabling a balanced comparison across the five absorber classes. The peak-frequency RMSE remains below 0.004 THz for all classes, while the spectral waveform R^2^ remains within the range of 0.9986–0.9996. The peak-frequency R^2^ ranges from 0.9862 to 0.9995, indicating that the model maintains high accuracy across different resonator topologies, although small topology-dependent variations remain. These results demonstrate that the proposed forward surrogate maintains consistently high predictive accuracy across predefined topological classes within the sampled design space. Representative spectral reconstructions for the five topology classes are provided in Supplementary Section S4 and Figure S3.

### 3.3. Ablation Study of Residual Architecture and Physics-Guided Loss

To isolate the contributions of the residual architecture and the proposed dynamic anchor loss, three forward models were evaluated using the same data split and preprocessing protocol. Model A uses a conventional multilayer perceptron with standard mean squared error (MSE); Model B uses the residual fully connected architecture with standard MSE; and Model C combines the same residual architecture with the dynamic anchor loss. Thus, the A–B comparison evaluates the contribution of residual connections, whereas the B–C comparison evaluates the contribution of the proposed loss. Statistical consistency across individual test samples was assessed using paired two-sided Wilcoxon signed-rank tests for the absolute resonance-frequency error and spectral RMSE, with rank-biserial correlation used as the effect-size measure and paired-bootstrap resampling used to estimate 95% confidence intervals.

As summarized in Table 3, introducing residual connections substantially improves resonance-frequency prediction. The mean peak-frequency RMSE decreases from 0.0259 THz for Model A to 0.0068 THz for Model B, corresponding to a 73.9% reduction. Incorporating the dynamic anchor loss further reduces the RMSE to 0.0027 THz, while the normalized frequency MSE decreases from 4.08 × 10^−4^ to 6.64 × 10^−5^ and the resonance-frequency R^2^ increases from 0.9804 to 0.9964. These results indicate that the residual architecture improves the nonlinear geometry–topology–response mapping, while the dynamic anchor loss provides additional supervision for resonance-centered features.

The paired sample-wise analysis confirmed that these improvements are statistically consistent. Model B significantly reduces the absolute resonance-frequency error relative to Model A (*p* < 0.001, *r_rb_* = 0.868), with a mean paired reduction of 0.0156 THz. Model C further improves upon Model B (*p* < 0.001, *r_rb_* = 0.764), yielding an additional mean reduction of 0.0033 THz. These results show that the resonance-frequency improvements associated with both the residual architecture and the dynamic anchor loss are consistently observed across individual test samples rather than arising solely from changes in aggregate MSE or RMSE values.

A different trend is observed at the waveform level. Model B yields a small but statistically significant reduction in the sample-wise spectral RMSE relative to Model A (*p* < 0.001, *r_rb_* = 0.223), whereas Model C shows a modest increase relative to Model B (*p* < 0.001, *r_rb_* = −0.459). This trade-off aligns with the multi-objective design of the dynamic anchor loss, which jointly constrains resonance position, local spectral shape, peak-center alignment, peak amplitude, and physical absorptivity bounds, rather than exclusively minimizing the conventional unweighted pointwise waveform error. Nevertheless, Model C retains high spectral fidelity, with a physical-domain local spectral RMSE of 0.0054 and topology-wise spectral R^2^ values of 0.9986–0.9996. The principal benefit of the proposed loss therefore lies in improved resonance-level physical accuracy while preserving high-quality spectral reconstruction.

**Figure 6:**
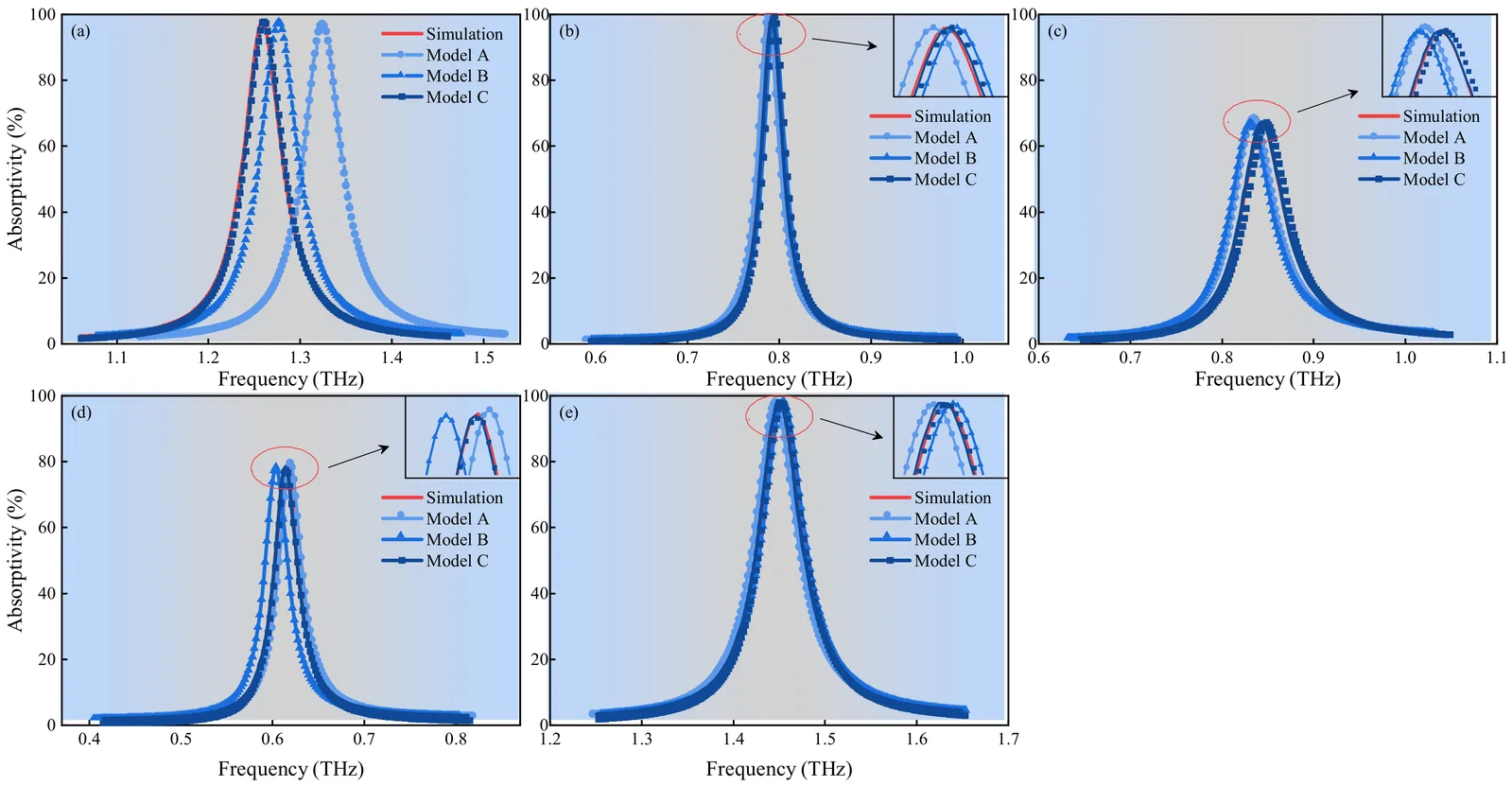
Topology-wise spectral comparison of the ablation models using identical geometric parameters. For each topology class, the absorption spectrum predicted by Model A (MLP + standard MSE), Model B (ResNet + standard MSE), and Model C (ResNet + dynamic anchor loss) is compared with the full-wave simulation spectrum using the same geometric parameters. Panels (**a**–**e**) correspond to Standard Cross, Jerusalem Cross, Cross-Enclosed Split Ring, Chiral Cross, and Split-Cross, respectively. The same geometric parameters are used for all three models within each topology class.

The topology-wise spectral comparisons in Figure 6 provide a direct visual confirmation of these improvements. For the Standard Cross example in Figure 6a, the resonance shift decreases from approximately 0.065 THz for Model A to 0.018 THz for Model B, while Model C provides the closest agreement with the full-wave spectrum in both peak position and amplitude. Similar improvements in resonance alignment are observed across the remaining topology classes. To further benchmark the complete resonance-anchored framework against conventional full-spectrum prediction, a full-spectrum ResNet baseline was trained using the same five-topology dataset and residual backbone with standard MSE loss. Relative to this baseline, Model C reduces the resonance-frequency RMSE from 0.0031 to 0.0027 THz and the 201-point local spectral RMSE from 0.0277 to 0.0059, corresponding to reductions of 12.9% and 78.7%, respectively. Together, these results demonstrate that the proposed framework improves resonance localization while maintaining accurate local spectral reconstruction across the predefined topology classes.

### 3.4. Physical Consistency and Model-Based Parameter Sensitivity

To examine whether the high predictive accuracy is accompanied by physically reasonable parameter dependence, the relationships between the geometric variables and resonance frequency were analyzed from both statistical and surrogate-model perspectives. First, Pearson correlation analysis was performed over the complete dataset to characterize the overall relationships among the continuous geometric parameters (*p*, *l*, *w*, *h*) and the resonance frequency (*f*_0_). Because the five topology classes occupy different geometric and frequency ranges, a topology-centered correlation analysis was further used to distinguish within-topology variations from topology-dependent offsets. Finally, input-gradient sensitivity analysis was conducted on the test set to quantify the local dependence of the trained surrogate on each continuous input variable.

**Figure 7:**
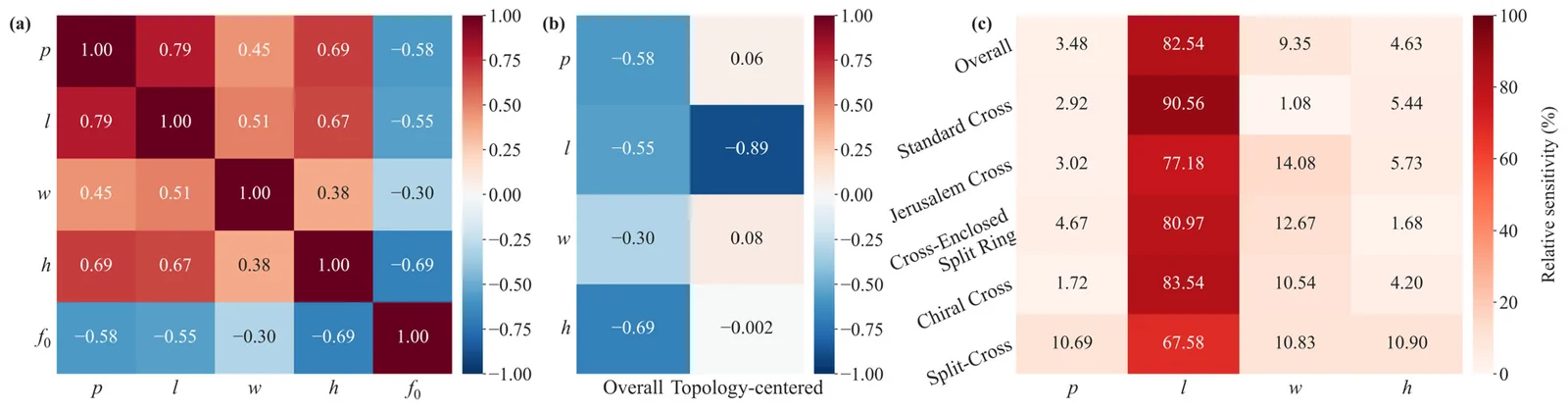
Statistical correlation and model-based sensitivity analyses of the resonance frequency with respect to the continuous geometric parameters. (**a**) Overall Pearson correlation matrix among the geometric parameters (*p*, *l*, *w*, *h*) and the resonance frequency (*f*_0_). (**b**) Comparison of overall and topology-centered Pearson correlations between the geometric parameters (*p*, *l*, *w*, *h*) and the resonance frequency (*f*_0_). (**c**) Relative input-gradient sensitivities of the model to the geometric parameters (*p*, *l*, *w*, *h*) for the test set and the five individual topology classes. The values in (**c**) are normalized within each row such that the contributions of the four continuous inputs sum to 100%.

As illustrated in Figure 7a, the overall Pearson analysis indicates negative correlations between resonance frequency (*f*_0_) and all four geometric parameters (*p*, *l*, *w*, *h*), with coefficients of −0.58, −0.55, −0.30, and −0.69, respectively. The geometric variables are also mutually correlated, for example *r_p_*_,*l*_ = 0.79, *r_p_*_,*h*_ = 0.69, and *r_l_*_,*h*_ = 0.67. Thus, over the complete multi-topology dataset, samples with larger characteristic dimensions generally occupy lower-frequency regions. However, because the five topology classes span different geometric and resonance-frequency ranges, these overall coefficients contain both within-topology parameter effects and systematic differences among topology classes. They should therefore not be interpreted directly as measures of the surrogate’s sensitivity to individual parameters.

To separate within-topology trends from topology-dependent offsets, the Pearson analysis was repeated after subtracting the topology-specific mean values of each geometric parameter and resonance frequency (*f*_0_). As shown in Figure 7b, the centered correlation between length (*l*) and resonance frequency (*f*_0_) is strongly negative (*r* = −0.885), whereas the corresponding coefficients for period (*p*), width (*w*) and thickness (*h*) are only 0.06, 0.08, and −0.002, respectively. Moreover, the *l*–*f*_0_ correlation remains strongly negative within each of the five topology classes, ranging from approximately −0.957 to −0.994. These results indicate that the relatively large overall correlations of period (*p*) and thickness (*h*) are strongly influenced by the different design regions occupied by the topology classes, whereas length (*l*) shows the most consistent relationship with resonance-frequency variation within individual topologies.

The statistical correlations describe the organization of the dataset but do not directly quantify how the trained surrogate depends on each input. Therefore, an input-gradient sensitivity analysis was performed on the test set by calculating the gradient of the predicted resonance frequency (*f*_0_) with respect to each standardized continuous variable. As shown in Figure 7c, the resonator length (*l*) exhibits the largest mean absolute sensitivity, 0.1663 THz per one-standard-deviation input change (95% CI: 0.1646–0.1679 THz), accounting for 82.54% of the total normalized sensitivity. The corresponding contributions of width (*w*), thickness (*h*) and period (*p*) are 9.35%, 4.63%, and 3.48%, respectively. Topology-wise results consistently identify length (*l*) as the dominant continuous parameter for all five classes.

The difference among Figure 7a–c is therefore not contradictory but reflects the different scopes of the analyses. The overall Pearson matrix characterizes the organization of the complete multi-topology dataset, the topology-centered analysis removes class-dependent offsets to reveal within-topology trends, and the gradient analysis quantifies the local input dependence learned by the surrogate. Taken together, the results show that period (*p*) and thickness (*h*) contribute to the separation of topology-dependent design regions, whereas the resonator length (*l*) is the most consistent within-topology control variable and the dominant continuous input governing the surrogate’s resonance-frequency prediction.

The dominant role of length (*l*) is physically consistent with the equivalent *RLC* description of the metal–dielectric–metal absorber. The resonance frequency can be approximated by Equation (3).

(3) $$f_{0}=1/2\pi \sqrt{LC}$$

where *L* and *C* denote the total equivalent inductance and capacitance, respectively. Under terahertz illumination, electromagnetic coupling between the patterned top layer and the metallic ground plane through the dielectric spacer produces a closed current loop and contributes to the magnetic resonance. The total capacitance includes contributions from the patterned-resonator coupling capacitance *C_g_* and interlayer capacitance *C_h_*, whereas the total inductance contains the self-inductance *L_m_* of the patterned metal and the current-loop contribution *L_h_*.

Increasing the resonator length l extends the surface-current path and increases the effective self-inductance *L_m_*, thereby increasing the *LC* product and shifting the resonance toward lower frequencies. This interpretation is consistent with both the strong negative topology-centered *l*–*f*_0_ correlation and the dominant input-gradient sensitivity learned by the surrogate. The remaining geometric parameters influence the resonance through different coupling mechanisms. The dielectric thickness (*h*) modifies the interlayer capacitance *C_h_* and the geometry of the magnetic current loop, whereas period (*p*) and width (*w*) affect inter-cell coupling, conductor geometry, and local capacitive interactions. Their contributions are therefore more topology-dependent, consistent with their weaker topology-centered correlations and lower surrogate sensitivities. Overall, the agreement among the topology-centered statistical trends, surrogate-gradient sensitivities, and equivalent-circuit interpretation indicates that the trained model captures a physically plausible and topology-consistent dependence of resonance frequency on the geometric parameters. This consistency provides further support for using the surrogate as a differentiable forward model in the subsequent inverse-design procedure.

### 3.5. PGD-Based Inverse Design and Comprehensive Verification

#### 3.5.1. PGD-Based Inverse Design and Full-Wave Verification

As demonstrated in the preceding sections, the trained forward surrogate model can accurately predict absorption spectra within the sampled design space. The next step is to determine whether the forward surrogate model can serve as a differentiable design engine for goal-oriented inverse design. To evaluate the capability of the proposed inverse-design framework, five inverse-design cases were constructed. The continuous geometric parameters were optimized to satisfy the target resonance frequency (*f_r_*), the target absorptivity (*A_r_*), and the target *Q*-factor (*Q_r_*). These target cases cover the low-frequency boundary response, attainable high absorption, topology-overlap behavior, stable mid-to-high-frequency matching, and a high-frequency-edge challenge. The targets were selected with reference to the resonance frequency, peak absorptivity, and Q-factor distributions of the training dataset. The corresponding response distributions and detailed target-selection rationale are provided in Supplementary Section S5 and Figure S4. The target responses used for inverse design are summarized in Table 4.

As illustrated in Table 4, the PGD-based inverse-design framework generates a series of candidate solutions that satisfy the target-response requirements, together with the corresponding predicted resonance characteristics, including the predicted resonance frequency (f_r_*), absorptivity (A_r_*), and *Q*-factor (Q_r_*). For T2–T4, the predicted resonance frequencies match the target values. Although minor deviations are observed for T1 and T5, their magnitudes range from 10^−3^ to 10^−4^ THz. The predicted absorptivity agrees well with the target values, particularly for T1, T2, and T4. For T5, the predicted peak absorptivity is 92.59%, which is slightly below the target value of 95%. This result indicates that simultaneously satisfying the resonance-frequency, peak-absorptivity, and quality-factor constraints becomes more challenging near the high-frequency edge of the data-supported response space. The larger discrepancy observed for T5 may be attributed to the increased sensitivity of high-frequency-edge designs and the greater uncertainty of the surrogate approximation near the boundary of the data-supported response domain. Full-wave electromagnetic verification is therefore particularly important for this case.

Furthermore, the optimal topology classes selected in Table 4 are consistent with the response regions inferred from the training data distribution. These topologies were not predefined but were selected by comparing the loss values across the five categories. For instance, the low-frequency boundary target T1 was ultimately assigned topology label 4, corresponding to Chiral Cross, while the high-frequency-edge target T5 was assigned topology label 5. This finding suggests that the PGD-based inverse-design model can automatically identify structurally suitable candidates for different spectral regions. T3 also exhibits a distinct one-to-many behavior. Although topology label 2 ultimately yielded the lowest loss, both labels 2 and 3 produced low-loss candidate solutions near the target frequency. This behavior is consistent with the overlapping frequency ranges of these two topology classes and confirms that different topological structures, combined with appropriate geometric parameters, can produce similar resonance responses. To further assess the sensitivity of the inverse-design procedure to the initial geometry, a multi-start PGD analysis was performed for all five target cases. Each topology was optimized from 10 different initial geometries, and the detailed initialization strategy and statistical results are provided in Supplementary Section S8 and Table S7. Overall, the multi-start search produced repeatable low-loss candidates, although the high-frequency boundary case T5 exhibited relatively greater sensitivity.

**Figure 8:**
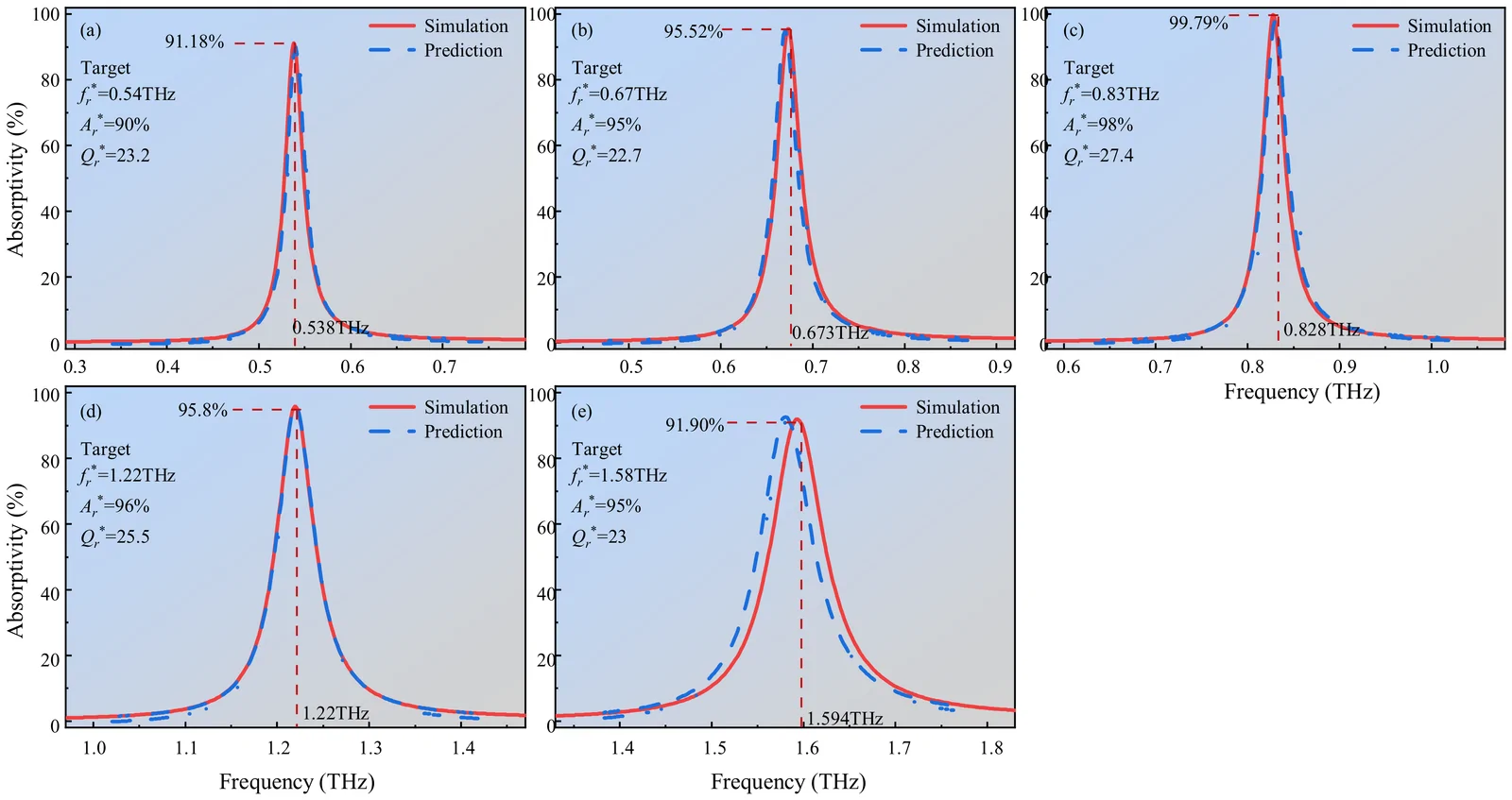
Full-wave simulation verification of the inverse-designed absorbers generated by PGD. The red solid curves represent the full-wave-simulated spectra of the optimized geometries, while the blue dashed curves represent the spectra predicted by the frozen forward surrogate model. Panels (**a**–**e**) correspond to the five inverse-design targets, T1–T5. The target resonance frequency, target absorptivity, and target quality factor are annotated in each panel.

To further validate the inverse-design capability of the model, the spectra obtained from full-wave electromagnetic simulations were compared with the surrogate-predicted spectra, as shown in Figure 8. Overall, the full-wave simulation spectra agree closely with the surrogate predictions. To further verify that the full-wave validation results are not sensitive to a particular numerical discretization, an additional mesh-convergence assessment was performed for all five inverse-designed absorbers. The corresponding spectra and quantitative convergence results are provided in Supplementary Section S7, Figure S5, and Table S6. This result indicates that the geometric parameters generated by the PGD-based inverse-design model retain their physical validity after full-wave electromagnetic verification. For T1–T4, the resonance peaks in the simulated spectra are located close to the target frequencies. T1 demonstrates the design capability at the low-frequency boundary, with the simulated resonance peak located at approximately 0.538 THz, differing from the target frequency by only 0.002 THz. T2 and T3 further demonstrate the model’s inverse-design capability to achieve high absorptivity in the low- to mid-frequency region and the overlapping-frequency region, with simulated peak absorptivity exceeding 95%. For T3, the peak absorptivity reaches 97.63%, indicating that the PGD-based inverse-design model can identify high-performance candidate solutions within regions of frequency overlap. T4 demonstrates that, at approximately 1.22 THz, the full-wave simulation results agree closely with the surrogate predictions, confirming the stability of inverse design within the mid-to-high-frequency range.

The largest surrogate-to-full-wave deviation occurs for T5. For the target *f_r_* = 1.58 THz and *A_r_* = 95%, the full-wave resonance occurs near 1.594 THz with a peak absorptivity of 91.90%. Because T5 was intentionally defined near the high-frequency boundary of the sampled response space, this discrepancy indicates reduced surrogate reliability near the edge of the data-supported domain. Although topology-specific projection and final full-wave verification mitigate this risk, boundary accuracy could be further improved through adaptive sampling in high-error regions and uncertainty quantification for low-confidence predictions. The T5 result therefore illustrates both the practical capability and the current boundary limitation of the surrogate-assisted inverse-design framework.

The proposed framework also differs from representative surrogate-learning and inverse-design approaches in the coordinated use of a resonance-anchored spectral representation, dynamic anchor loss, and frozen-surrogate gradient optimization. By explicitly predicting the dominant resonance frequency together with the resonance-centered local spectrum, the framework directly emphasizes resonance-sensitive features during forward modeling. The validated forward surrogate is then reused as a frozen differentiable model for topology-wise PGD, avoiding the need to train a separate inverse network. A detailed methodological comparison with representative recent approaches is provided in Supplementary Section S6 and Table S5.

#### 3.5.2. Electromagnetic Field and Surface-Current Analysis

**Figure 9:**
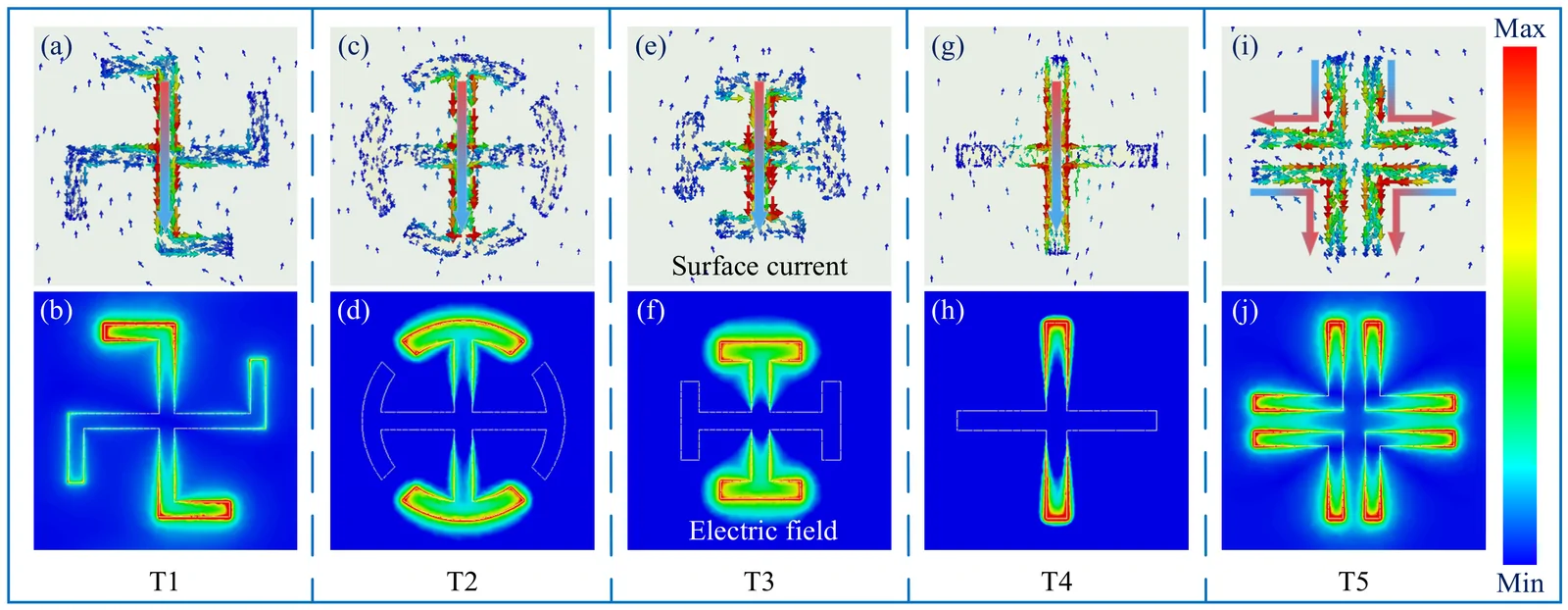
Surface-current and electric-field distributions of the inverse-designed absorbers at their respective resonance frequencies. (**a**,**b**) T1, Chiral Cross at 0.54 THz; (**c**,**d**) T2, Cross-Enclosed Split Ring at 0.67 THz; (**e**,**f**) T3, Jerusalem Cross at 0.83 THz; (**g**,**h**) T4, Standard Cross at 1.22 THz; and (**i**,**j**) T5, Split-Cross at 1.58 THz. Panels (**a**,**c**,**e**,**g**,**i**) show the surface-current distributions on the patterned top metallic layer, while panels (**b**,**d**,**f**,**h**,**j**) show the corresponding electric-field distributions. The field magnitudes are normalized independently to emphasize the spatial resonance modes.

To further elucidate the physical origin of the resonance responses predicted for the inverse-designed absorbers, the surface-current and electric-field distributions of the five designs T1–T5 were analyzed at their respective resonance frequencies, as shown in Figure 9. The absorbers employ a metal–dielectric–metal configuration, in which the patterned top resonator and metallic ground plane are separated by a dielectric spacer. This configuration forms a Fabry–Pérot cavity, within which the incident electromagnetic waves undergo multiple reflections between the metallic layers and generate standing-wave distributions. These standing waves enhance the electromagnetic field within the dielectric spacer and contribute to the excitation of magnetic dipole resonances. At resonance, the induced currents on the two metallic layers provide direct evidence of this magnetic response. For T1–T4, the dominant current on the ground plane flows opposite to that on the patterned top layer, forming a circulating current loop through the metal–dielectric–metal configuration and thereby supporting a magnetic dipole resonance. T5 exhibits a more localized current-loop configuration because the Split-Cross can be regarded as four coupled L-shaped resonant elements. For a representative L-shaped element, the current on the top layer propagates from one end of the bent metallic path to the other, whereas the corresponding ground-plane current flows from the terminal region back toward the initial region. The oppositely directed currents therefore form a closed loop and generate a similar magnetic response. For clarity, only the surface currents on the patterned top metallic layers are displayed in Figure 9.

The electric-field and top-layer current distributions further reveal the topology-dependent localized electric resonances. For T1 (Figure 9a,b), strong electric fields are localized mainly at the ends and bends of the Chiral Cross arms, whereas the surface current follows the elongated and folded metallic paths. The extended current path increases the effective inductive contribution, while the multiple bends and terminal regions provide distributed capacitive coupling, consistent with the relatively low resonance frequency of approximately 0.54 THz. For T2 (Figure 9c,d), the field is strongly enhanced around the upper and lower curved sections and their coupling regions with the central cross, while the current extends over both the central resonator and the surrounding split-ring sections. This distribution indicates a coupled resonance involving the central cross and the surrounding split-ring elements. T3 (Figure 9e,f) exhibits strong field localization near the terminal sections of the Jerusalem Cross and pronounced currents along the central stem and extended branches, indicating that the additional branches modify both the effective current path and local capacitive coupling. In T4 (Figure 9g,h), the current is predominantly concentrated along the longitudinal arm of the Standard Cross, while the electric field is strongly localized at its upper and lower ends, representing a comparatively simple dipole-like localized resonance. In contrast, T5 (Figure 9i,j) consists of several shorter and more localized current paths associated with the split L-shaped elements. Strong electric fields appear near the split gaps and metallic edges, indicating multiple localized capacitive-coupling regions. The shorter effective current paths are consistent with its higher resonance frequency near 1.58 THz.

Although the five absorber topologies exhibit distinct spatial field patterns, they share a common physical picture. The Fabry–Pérot cavity formed by the metal–dielectric–metal configuration establishes the standing-wave field distribution, while the enhanced electric fields at metallic ends, gaps, bends, and adjacent edges indicate localized charge accumulation and capacitive coupling. The dominant surface-current paths provide the corresponding inductive contribution. In combination with the oppositely directed top- and ground-plane currents, these localized electric resonances couple with the magnetic dipole resonance generated by the standing-wave excitation in the cavity to produce the strong absorption peaks.

#### 3.5.3. Robustness Analysis Under Geometric Perturbations

To further evaluate the robustness of the inverse-designed absorbers against possible fabrication-induced dimensional deviations, a manufacturing-tolerance analysis was performed for all five investigated topology classes. For each topology, the inverse-designed geometry was taken as the nominal design, and representative relative dimensional perturbations within ±4% were introduced into the optimized geometric parameters. In addition to the nominal (0%) design and the ±4% boundary cases, one intermediate negative perturbation and one intermediate positive perturbation were considered for each topology. All perturbed geometries were recalculated using full-wave simulations.

**Figure 10:**
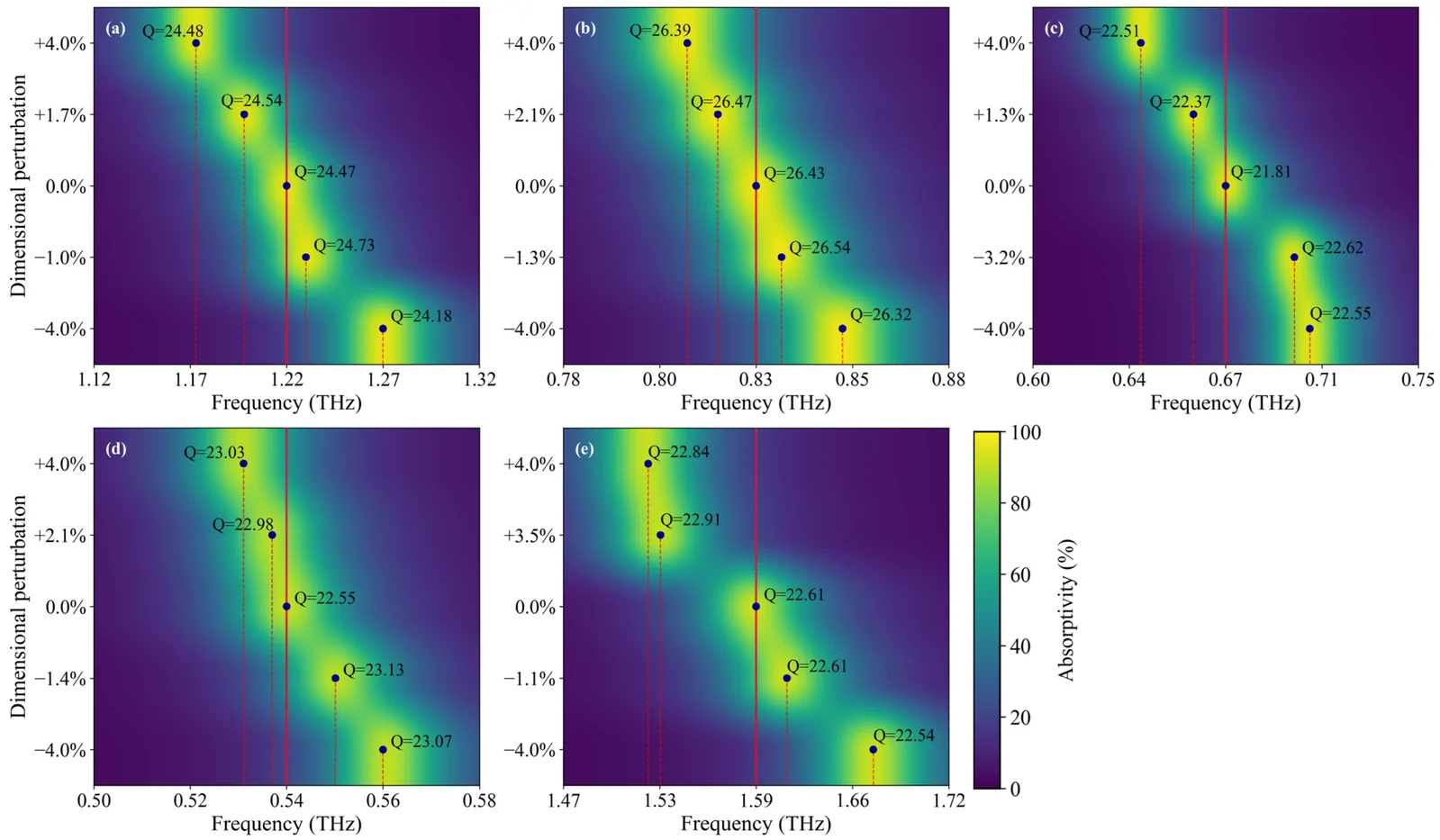
Manufacturing-tolerance response maps of the five inverse-designed terahertz metamaterial absorbers under representative dimensional perturbations within ±4%. (**a**) Standard Cross; (**b**) Jerusalem Cross; (**c**) Cross-Enclosed Split Ring; (**d**) Chiral Cross; and (**e**) Split-Cross. The color scale represents absorptivity, the markers indicate the dominant resonance frequencies, and the corresponding Q-factors are annotated. The red solid line denotes the nominal resonance frequency of the 0% design, while the red dashed lines indicate the resonance-frequency positions under the perturbed geometries.

Figure 10a–e presents the corresponding tolerance-response maps for the five absorber topologies. A systematic shift in the dominant resonance frequency is observed as the structural dimensions vary, whereas the peak absorptivity and *Q*-factor remain comparatively stable. Table 5 quantitatively summarizes the nominal resonance characteristics and the maximum deviations induced by the considered dimensional perturbations. Across the five topology classes, the maximum relative resonance-frequency deviation is approximately 4.90%, the maximum variation in peak absorptivity is approximately 1.90 percentage points, and the maximum relative variation in the *Q*-factor is approximately 3.70%.

Although topology-dependent differences in sensitivity are observed, all five inverse-designed absorbers preserve their dominant resonance characteristics within the considered ±4% dimensional-perturbation range. These results indicate that the proposed inverse-designed structures exhibit reasonable robustness against representative fabrication-induced geometric deviations, with the principal effect of dimensional perturbation being a shift in resonance frequency rather than a substantial degradation of peak absorptivity or *Q*-factor.

## 4. Conclusions

This study presents a resonance-anchored residual surrogate framework for forward prediction and inverse design across multiple predefined terahertz metamaterial absorber topologies. By separating the dominant resonance frequency from the local spectral profile and incorporating a physics-guided dynamic anchor loss, the proposed surrogate improves resonance localization while maintaining high spectral-reconstruction accuracy. Ablation analysis confirms the respective contributions of the residual architecture and resonance-aware loss formulation. The validated forward surrogate is further reused as a frozen differentiable model for topology-wise PGD inverse design. Full-wave simulations confirm that the optimized candidates reproduce the prescribed resonance responses with good accuracy within the data-supported design domain, while the high-frequency boundary case shows increased sensitivity to surrogate approximation error. Multi-start, mesh-convergence, and geometric-tolerance analyses further support the robustness of the resulting design workflow. The present model is intended for the topology classes and parameter ranges represented in the training data. Extension to broader parameter spaces, additional materials, or more complex geometries would require corresponding expansion of the structural representation and training dataset, followed by retraining and validation.

## Figures and Tables

**Table 1:** Distribution of Design Parameters for Five Types of Metamaterial Absorbers.

Parameters	Standard Cross	Jerusalem Cross	Cross-Enclosed Split Ring	Chiral Cross	Split-Cross
period (*p*/μm)	90–108	100–118	94–112	132–150	95–113
length (*l*/μm)	62–80	62–80	66–84	92–110	67–85
width (*w*/μm)	7–10	5–8	6–9	8–11	5–8
thickness (*h*/μm)	4–8	6–10	8–12	11–15	5–9

**Table 2:** Topology-wise predictive performance on the test set.

Topology Structure Label	Count	Peak RMSE/THz	Peak R^2^	Amplitude Max Error	Spectral RMSE	Spectral R^2^
1	200	0.0033	0.9988	0.0171	0.0054	0.9994
2	200	0.0018	0.9985	0.0307	0.0066	0.9989
3	200	0.0018	0.9995	0.0119	0.0062	0.9991
4	200	0.0027	0.9862	0.0170	0.0066	0.9986
5	200	0.0038	0.9991	0.0278	0.0047	0.9996

**Table 3:** Ablation comparison of forward surrogate models on the blind test set.

Model	Architecture	Loss Function	Frequency MSE	Spectral MSE	Mean Peak RMSE/THz	Mean Peak R^2^
Model A	MLP	Standard MSE	6.72 × 10^−3^	3.74 × 10^−3^	0.0259	0.8466
Model B	ResNet	Standard MSE	4.08 × 10^−4^	3.25 × 10^−3^	0.0068	0.9804
Model C	ResNet	Dynamic anchor loss	6.64 × 10^−5^	3.58 × 10^−3^	0.0027	0.9964

**Table 4:** PGD-generated candidate geometries.

Target Case	Target			Topology Label	Optimized Geometry (μm)	Predicted			Inverse Design Loss
	f_r_* (THz)	A_r_*	Q_r_*			${\hat{f}}_{r}$ (THz)	${\hat{A}}_{r}$	${\hat{Q}}_{r}$	
T1	0.54	90%	23.2	4	*p* = 150.00; *l* = 110.00; *w* = 8.44; *h* = 14.29	0.5402	90.02%	22.89	2 × 10^−6^
T2	0.67	95%	22.7	3	*p* = 106.98; *l* = 80.80; *w* = 7.22; *h* = 10.59	0.6700	95.00%	22.54	0.00
T3	0.83	98	27.4	2	*p* = 118.00; *l* = 70.12; *w* = 8.00; *h* = 9.08	0.8300	98.16%	27.03	3.7 × 10^−5^
				3	*p* = 112.00; *l* = 71.37; *w* = 8.98; *h* = 8.20	0.8303	98.85%	26.86	1.04 × 10^−3^
T4	1.22	96%	25.5	1	*p* = 94.69; *l* = 70.35; *w* = 7.14; *h* = 6.18	1.2200	96.00%	25.14	0.00
T5	1.58	95%	23.0	5	*p* = 95.00; *l* = 70.50; *w* = 5.00; *h* = 6.41	1.5806	92.59%	23.35	1.07 × 10^−2^

**Table 5:** Manufacturing-tolerance performance of the five inverse-designed absorber topologies.

Topology Label	*f*_0_ (THz)	Δ*f*_0_ (%)	*A* (%)	Δ*A* (pp)	*Q*	Δ*Q* (%)
1	1.220	4.10	95.80	0.53	24.47	1.22
2	0.829	2.70	97.63	0.73	26.43	0.43
3	0.673	**4.90**	95.52	0.74	21.81	**3.70**
4	0.538	3.72	91.18	**1.90**	22.55	2.60
5	1.594	4.77	91.90	0.57	22.61	1.35

Bold values indicate the largest variation among the five absorber topologies for each manufacturing-tolerance metric, representing the worst-case sensitivity to dimensional perturbations.

## Data Availability

The original contributions presented in this study are included in the article. Further inquiries can be directed to the corresponding author.

## Supplementary Materials

The following supporting information can be downloaded at: https://www.mdpi.com/article/10.3390/nano16171107/s1, Figure S1: Architecture of the residual fully connected forward prediction network; Table S1: Definitions and weights of the dynamic anchor loss; Table S2: Forward-surrogate architecture and training hyperparameters; Table S3: PGD inverse-design objective, projection rule, and optimization settings; Figure S2: Training and validation loss curves of the forward surrogate model; Table S4: Prediction performance of Model C across ten independent training runs; Figure S3: Representative spectral reconstruction across five topology classes. (a) Standard Cross, (b) Jerusalem Cross, (c) Cross-enclosed Split Ring, (d) Chiral Cross, and (e) Split-Cross. Red solid lines denote simulated spectra, and blue dashed lines denote predicted spectra; Figure S4: Training-set response distributions used for inverse-design target selection; Table S5: Methodological comparison of representative recent surrogate-learning and inverse-design approaches for metamaterial and metasurface absorbers; Figure S5: Mesh-convergence comparison of the absorption spectra for the five inverse-designed absorber cases. (a) T1; (b) T2; (c) T3; (d) T4; and (e) T5. For each optimized geometry, the material properties, excitation, boundary conditions, and electromagnetic solver were kept unchanged, while the numerical mesh was progressively refined using mesh settings of 10, 15, and 20. The spectra obtained using settings 15 and 20 show close agreement for all five cases, indicating numerical convergence of the principal resonance characteristics after sufficient mesh refinement. The high-frequency-edge T5 case exhibits a larger resonance-frequency shift for the coarsest mesh setting, but the response becomes stable with further refinement; Table S6: Mesh-convergence results for the five inverse-designed absorbers; Table S7: Multi-start robustness of PGD inverse design for T1–T5.
