# Supplementary material for: Resonance-Anchored Residual Surrogate for Spectral Prediction and Inverse Design of Multiple Terahertz Metamaterial Absorber Topologies
Source: Nanomaterials (Basel). 2026 Sep 3;16(17):1107. doi: 10.3390/nano16171107 (PMC13567527; doi:10.3390/nano16171107)
Supplement: Supplementary file 1 [file nanomaterials-16-01107-s001.zip › nanomaterials-4485537-supplementary.pdf]

Supplementary File

Resonance-Anchored Residual Surrogate for Spectral Prediction and Inverse Design of Multiple Terahertz Metamaterial Absorber Topologies

Guilin Li, Weiwei Qu, Yan Huang, Yurong wang, Qin Yi, and Hu Deng *

School of Information and Control Engineering, Southwest University of Science and Technology, Mianyang 621010, China
* Correspondence: denghu@swust.edu.cn

Section S1. Forward surrogate architecture and dynamic loss formulation

The forward surrogate network is based on the Residual Neural Networks (ResNet) architecture, the model receives a 9-dimensional input vector consisting of four continuous geometric parameters and five one-hot encoded topology labels. The input is mapped into a 512-dimensional latent space, followed by eight residual blocks with skip connections. The final output is a 202-dimensional spectral response, including one predicted resonant center frequency and a 201-point local absorption spectrum. The forward surrogate core components include an initial feature mapping layer, eight successive residual blocks, and a linear output layer. Figure S1 demonstrates that the ResNet model begins with an initial layer that upsamples input data from a low-dimensional physical feature space to a higher-dimensional feature space. This section involves a sequence of a fully connected layer, batch normalization, and a GELU activation function. The residual blocks facilitate the extraction of deep-level features. Inside the model, the structure consists of a fully connected layer with 512 neurons, a batch normalization layer, a GELU activation, and a dropout layer, followed by a second fully connected layer with 512 neurons, another batch normalization layer, and a dropout layer. Meanwhile, each module uses a skip connection to combine the input features with the output of the main branch. These combined features are then processed using a nonlinear GELU activation. After thorough feature extraction with eight residual blocks, the resulting feature data is forwarded to the output layer, which consists of fully connected layers.

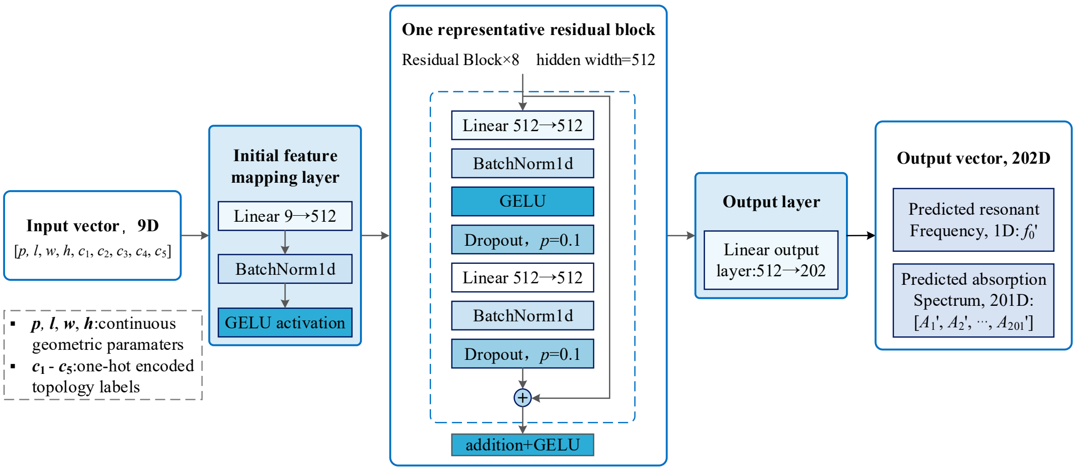

Figure S1. Architecture of the residual fully connected forward prediction network.

In the prediction task of this article, since the output vector contains a scalar resonant frequency and 201 spectral amplitude values, using the standard mean square error loss is not sufficiently comprehensive. When all dimensions are averaged uniformly, the high-dimensional waveform error can dominate the optimization, while the low-dimensional but physically critical resonant frequency may be underweighted. In addition, for narrowband terahertz absorbers, a small horizontal displacement of the resonance peak can correspond to a meaningful physical frequency shift, even when the average pointwise spectral error is relatively small. When

used as a loss function, traditional MSE is computed by taking the mean over the feature dimensions. The absorption spectral errors generate dominant accumulated gradients during backpropagation, thereby diminishing the significance of resonance frequencies. Therefore, to address these issues, a physics-guided dynamic anchor loss was adopted. The total loss function is defined as shown below:

$$\mathcal{L} = \omega_f \mathcal{L}_f + \omega_s \mathcal{L}_{\text{spec}} + \omega_c \mathcal{L}_{\text{center}} + \omega_a \mathcal{L}_{\text{amp}} + \omega_b \mathcal{L}_{\text{bound}} \quad \text{(S1)}$$

Here $\mathcal{L}_f$, $\mathcal{L}_{\text{spec}}$, $\mathcal{L}_{\text{center}}$, $\mathcal{L}_{\text{amp}}$ and $\mathcal{L}_{\text{bound}}$ represent the resonant-frequency loss, local spectral waveform loss, peak-center alignment loss, peak-amplitude loss, and absorptivity-boundary loss, respectively. The coefficients ω_f, ω_s, ω_c, ω_a and ω_b are used to balance the contributions of each term during training; the specific meanings of each loss term and their weight values are shown in **Table S1**.

Table S1. Definitions and weights of the dynamic anchor loss

Loss Function	Mathematical Definition	Meaning	Weight
$\mathcal{L}_f$	$\frac{1}{N}\sum_{i=1}^{N}(\hat{f}_{0,i}^{n} - f_{0,i}^{n})^2$	Constrained Resonance Center Frequency	$\omega_f = 50$
$\mathcal{L}_{\text{spec}}$	$\frac{1}{NM}\sum_{i=1}^{N}\sum_{j=1}^{M} m_{i,j}(\hat{A}_{i,j}^{n} - A_{i,j}^{n})^2$	Constrained Local Spectrum Shapes	$\omega_s = 1$
$\mathcal{L}_{\text{center}}$	$\frac{1}{N}\sum_{i=1}^{N}(\hat{I}_i - I_c)^2$	Suppressing Offset in Predicted Peak Position	$\omega_c = 15$
$\mathcal{L}_{\text{amp}}$	$\frac{1}{N}\sum_{i=1}^{N}(\hat{I}_i - I_c)^2$	Constrained Peak Absorption Rate	$\omega_a = 10$
$\mathcal{L}_{\text{bound}}$	$\frac{1}{NM}\sum_{i=1}^{N}\sum_{j=1}^{M}\left[\max(0, \hat{A}_{i,j}^{phys} - 1)^2 + \max(0, -\hat{A}_{i,j}^{phys})^2\right]$	Ensure that the absorption rate falls within the physical range	$\omega_b = 10$

The first and second loss terms constrain the resonant center frequency of the network's predictions and the local absorption profile around the resonance peak, respectively.

$$\mathcal{L}_f = \frac{1}{N}\sum_{i=1}^{N}(\hat{f}_{0,i}^{n} - f_{0,i}^{n})^2 \quad \text{(S2)}$$

$$\mathcal{L}_{\text{spec}} = \frac{1}{NM}\sum_{i=1}^{N}\sum_{j=1}^{M} m_{i,j}(\hat{A}_{i,j}^{n} - A_{i,j}^{n})^2 \quad \text{(S3)}$$

where $m_{i,j}$ is a spectral weighting mask used to emphasize the resonance-related region, giving more attention to the absorption peak rather than being dominated by flat off-resonance regions

Although it constrains the absorption spectrum, it does not guarantee that the predicted peak will always be located at the center of the window anchored by the resonance peak. Thus, to suppress peak shifts, a differentiable peak center alignment loss function is introduced. First, the predicted normalized spectrum is transformed back into the physical absorbance space:

$$\hat{A}_{i,j}^{phys} = \hat{A}_{i,j}^{n}\sigma_j + \mu_j \quad \text{(S4)}$$

where μ_j and σ_j are the mean and standard deviation of the j-th spectral point, computed from the training set. The Soft-Argmax operation is then applied to estimate the peak location in a differentiable manner and the Soft-Argmax inverse-temperature coefficient was set to β=20.

$$\hat{I}_i = \sum_{j=1}^{M} j \frac{\exp(\beta \hat{A}_{i,j}^{phys})}{\sum_{k=1}^{M}\exp(\beta \hat{A}_{i,k}^{phys})} \quad \text{(S5)}$$

The peak-center alignment loss explicitly penalizes horizontal shifts of the predicted absorption peak, thereby improving the physical consistency of the local spectral reconstruction. This loss term is given by

$$\mathcal{L}_{\text{center}} = \frac{1}{N}\sum_{i=1}^{N}(\hat{I}_i - I_c)^2 \quad \text{(S6)}$$

In addition to the peak position, the peak absorptivity is a key performance indicator for metamaterial absorbers. A model may reproduce the overall spectral shape yet still underestimate or overestimate the resonant absorption amplitude. Therefore, a peak-amplitude loss is added to directly constrain the height of the predicted resonance peak:

$$\mathcal{L}_{\text{amp}} = \frac{1}{N}\sum_{i=1}^{N}(\hat{A}_i^{peak} - A_{i,I_c}^{phys})^2 \quad \text{(S7)}$$

In this equation, $\hat{A}_i^{peak}$ denotes the predicted absorptivity at the differentiable peak location estimated by the Soft-Argmax operation, and A_{i,I_c}^{phys} is the true absorptivity at the center of the local resonance-anchored window.

The final constraint limits absorptivity to the range [0, 1], ensuring that the predicted absorption response adheres to physical principles. However， the forward surrogate network uses a linear output layer and therefore does not enforce this constraint. To address this issue, an absorptivity boundary loss is implemented within the physical absorptivity space.

$$\mathcal{L}_{\text{bound}} = \frac{1}{NM}\sum_{i=1}^{N}\sum_{j=1}^{M}\left[\max(0, \hat{A}_{i,j}^{phys} - 1)^2 + \max(0, -\hat{A}_{i,j}^{phys})^2\right] \quad \text{(S8)}$$

This term penalizes only out-of-range predictions and leaves spectral points that already satisfy the physical bound $0 \le \hat{A}_{i,j}^{phys} \le 1$ unchanged. Therefore, it serves as a soft physical constraint on the model's output without altering the network's regression nature.

In the above equations, N represents the batch size, M=201 represents the number of local spectral sampling points, f_0 and $\hat{f}_0$ represent the true and predicted resonance frequencies, respectively. $A_{i,j}$ and $\hat{A}_{i,j}$ represent the true and predicted absorptivity, respectively. The superscripts n and $phys$ denote the standardized space and the inverse-normalized physical absorptivity space, respectively. I_c is the center index of the local spectral window.

The complete dataset was divided into training, validation, and independent blind test sets using a stratified 8:1:1 split. Meanwhile, to prevent data imbalance caused by random distribution, a stratified sampling strategy was employed, ensuring that each topological structure strictly adhered to this ratio across all subsets. Additionally, to reduce the impact of dimensional and order-of-magnitude differences in the training data on model performance, all structural parameters, as well as the frequency and intensity values of the absorption spectrum, were normalized using Z-scores to be in a standard normal distribution with a mean of 0 and a standard deviation of 1 before training.

$$z_i = \frac{x_i - \mu}{\sigma} \quad \text{(S9)}$$

where x_i denotes the values in the original data sample, whereas z_i represents the values subsequent to standardization. The symbols μ and σ denote the mean and standard deviation of the feature across the entire training set, respectively.

The model was trained using the AdamW optimizer with a learning rate of 0.0001 and a batch size of 128. To enhance convergence and prevent overfitting, a cosine-annealing warm-restart scheduler and early-stopping strategy were employed. All neural-network models were trained on a laptop equipped with an NVIDIA GeForce RTX 3050 GPU, a 12th Gen Intel(R) Core(TM) i5-12500H processor, and 32 GB of system memory. The implementation used Python 3.8. The proposed model required approximately 23 min of wall-clock time for a complete training run. For the primary model reported in the main manuscript, the early-stopping criterion was triggered at epoch 1208. Forward-surrogate architecture and training hyperparameters are shown in **Table S2** All trained model parameters and fitting scalers were saved for later testing and inverse design. During training, the model's forward prediction accuracy was assessed through the normalized MSE for resonant frequency and spectral waveforms. Post-inverse normalization, evaluation included physically meaningful metrics like peak-frequency RMSE, R^2, spectral RMSE, spectral waveform R^2, and maximum peak-amplitude error.

Table S2. Forward-surrogate architecture and training hyperparameters

Hyperparameters	Content
Input dimension	9

Hyperparameters	Content
Continuous geometry variables	4
Topology encoding dimension	5
Hidden dimension	512
Residual blocks	8
Output dimension	202
Activation	GELU
Normalization	Batch normalization
Optimizer	AdamW
Learning rate	1×10^{-4}
Batch size	128
Scheduler	cosine annealing with warm restarts
Soft-Argmax inverse temperature	β=20
Early stopping	Yes
Final retained epoch of the primary model	1208
GPU	RTX 3050
CPU	i5-12500H
RAM	32 GB
Python	3.8
Training time	approximately 23 min per complete run

Section S2. PGD inverse-design objective and projection

After the forward surrogate model has been trained and validated, it is frozen and embedded into a gradient-based inverse design loop. Unlike inverse neural networks that map from spectrum to structure, this approach treats continuous geometric parameters as optimizable variables and updates them through backpropagation while keeping all network weights fixed.

The geometric parameters x = [p, l, w, h] are treated as continuous optimization variables, whereas the topology class represents a discrete structural configuration. Therefore, the topology label is not optimized through gradient backpropagation. Instead, the five topology classes are searched independently using fixed one-hot labels. For each topology, PGD is performed from 10 independent initial geometries. The first restart is initialized using the topology-specific mean geometry of the training samples, whereas the remaining nine initial geometries are uniformly sampled within the corresponding topology-specific feasible bounds. All restarts use the same objective function, learning rate, number of optimization steps, and projection constraints. After convergence, the candidates obtained from all topology–restart combinations are ranked according to their final inverse-design loss, and the best-performing candidate is retained for subsequent verification. This topology-wise multi-start strategy reduces dependence on a single initial geometry while preserving the discrete nature of the topology variable.

To impose constraints on the position, intensity, and bandwidth of the absorption peak, the inverse design objective function is defined as follows for each fixed topology:

$$\mathcal{L}_{inv} = \lambda_f(\hat{f}_r - f_r^*)^2 + \lambda_A(\hat{A}_r - A_r^*)^2 + \lambda_Q[\log(\hat{Q}_r + \varepsilon) - \log(Q_r^* + \varepsilon)]^2 + \lambda_b\mathcal{L}_b \tag{S10}$$

where f_r^*, A_r^*, Q_r^* denote the target resonant frequency, target resonant absorptivity, and target quality factor, respectively. The quantities that are predicted by the frozen forward surrogate are denoted by $\hat{f}_r$, $\hat{A}_r$, and $\hat{Q}_r$. The initial term is responsible for directing the optimized structure towards the desired resonant frequency, while the second term restricts the absorption intensity at resonance. The third term is responsible for the modulation of both the sharpness and the bandwidth of the resonance, a process that is known as the quality factor. In this work, the quality factor is defined as $Q = \frac{f_r}{\mathrm{FWHM}}$, where FWHM is the full width at half maximum of the absorption

peak. The introduction of a small constant, designated as ε, has been shown to circumvent numerical instability when the predicted Q approaches zero. The expression of the Q term in logarithmic form has been demonstrated to be effective in the mitigation of both relative deviations in resonance sharpness and numerical imbalances caused by disparate orders of magnitude of the Q factor, frequency, and absorption coefficient.

The term $\mathcal{L}_b = \sum_d [\max(0, x_d - x^c_{d,\max})^2 + \max(0, x^c_{d,\min} - x_d)^2]$ denotes a boundary penalty that discourages geometries that violate the topology-specific feasible parameter ranges. Here, x_d is the d-th component of the design vector x, $x^c_{d,\max}$ and $x^c_{d,\min}$ are the lower and upper bounds of this parameter for the topology. This term provides a soft feasibility constraint, whereas the projection operator in PGD enforces the hard boundary after each update. The coefficients λ_f, λ_A, λ_Q and λ_b, balance the contributions of frequency matching, absorptivity matching, quality-factor matching, and geometric feasibility, respectively.

Subsequently, the geometric parameters are refined using the Projected Gradient Descent (PGD) procedure. The inverse-design loss is evaluated through the frozen forward surrogate, and backpropagation computes gradients with respect to the input geometric parameters. After each gradient update, the parameters are projected back into the topology-specific feasible region. This procedure is repeated independently for all topology classes and restart initializations. The geometric parameters are updated according to the PGD formulation given below:

$$x_{t+1} = \Pi_{\Omega_c}(x_t - \eta \nabla x_t \mathcal{L}_{inv}(f_\theta(x_t, c), y^*)) \tag{S11}$$

where x_t represents the geometric parameters of the metamaterial absorber configuration (x = [p, l, w, h]), η represents the backward optimization learning rate, f_θ represents the forward prediction model with fixed weights, y^* represents the target response, and Π_{Ω_c} represents the constraint projection operator, which maps parameters that exceed physical boundaries back into the feasible region. Based on the constraints among the various geometric parameters, the mathematical expression for the constraint projection operator in this study is:

$$\Pi_{\Omega_c}(x) = \min(x_{\max}, \max(x_{\min}, x)) \tag{S12}$$

After convergence, each topology–restart combination yields a candidate geometry. All candidates are ranked according to the final inverse-design loss, and the best-performing topology–geometry combination obtained from the multi-start search is selected for full-wave verification. When multiple candidates exhibit comparable losses, the top-k solutions can be retained to reflect the one-to-many nature of the inverse problem. The selected candidate is subsequently reconstructed in the full-wave electromagnetic model for independent spectral verification. The full-wave simulation spectrum is compared with both the target response and the surrogate-predicted response using the resonance-frequency deviation, peak-amplitude error, and local spectral RMSE. **Table S3**. shows the PGD inverse-design objective, projection rule, and optimization settings.

Table S3. PGD inverse-design objective, projection rule, and optimization settings

Project	Definition/Settings
Optimized variables	x=[p,l,w,h]
Topology treatment	Five types of topologies are optimized independently using fixed one-hot labels
Initialization	10 starts per topology: one topology-specific mean initialization and nine uniformly sampled random initializations within the corresponding feasible bounds
Number of restarts	10 per topology
Target quantities	f_r^*, A_r^*, Q_r^*

Objective	$\lambda_f(\hat{f}_r - f_r^*)^2 + \lambda_A(\hat{A}_r - A_r^*)^2 + \lambda_Q[\log(\hat{Q}_r + \varepsilon) - \log(Q_r^* + \varepsilon)]^2 + \lambda_b \mathcal{L}_b$
Coefficient	λ_f=50; λ_A=10; λ_Q=5; λ_b=11
Boundary penalty	$\sum_d [\max(0, x_d - x_{d,max}^c)^2 + \max(0, x_{d,\min}^c - x_d)^2]$
Projection rule	$\Pi_{\Omega_c}(x) = \min(x_{\max}, \max(x_{\min}, x))$
Feasible bounds	topology-specific parameter ranges
Candidate selection	Lowest-loss candidate among all topology–restart combinations after convergence
Verification	full-wave simulation

Section S3. Training convergence and reproducibility assessment

Section S3.1 Training convergence

Figure S2 shows the training and validation loss curves of the proposed forward prediction network. Both losses decrease rapidly during the early stage of training, indicating that the residual network can efficiently capture the nonlinear mapping between geometric parameters, topology labels, and absorption spectral responses. Periodic fluctuations in the loss curves are observed during training. These oscillations are consistent with the cosine annealing with warm restarts schedule, in which the learning rate is periodically increased to promote exploration of the loss landscape. The early stopping criterion was triggered at epoch 1208, and the model weights corresponding to the lowest validation loss were retained for subsequent blind-test evaluation.

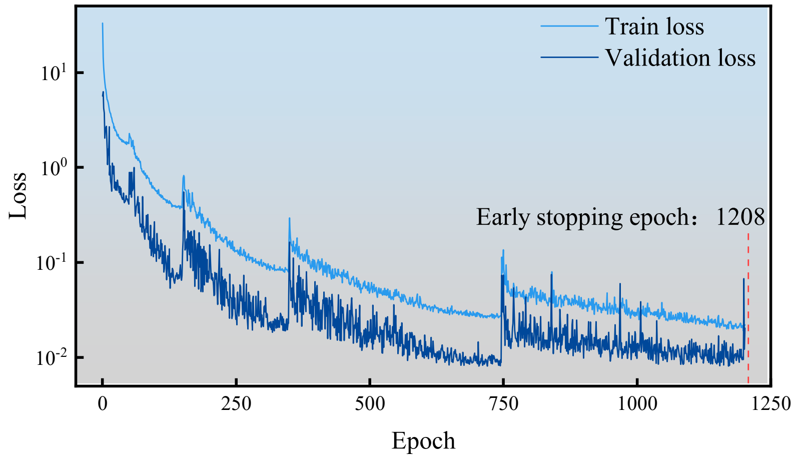

Figure S2. Training and validation loss curves of the forward surrogate model.

Section S3.2 Reproducibility across independent training runs

To further assess whether the predictive performance of Model C depends on a specific random train–validation–test partition or network initialization, ten additional independent training runs were conducted. For each run, a distinct random seed was used to regenerate the topology-stratified 8:1:1 train–validation–test split. The input and output standardization parameters were refitted using only the training subset generated for that run, and the network was trained from a new random initialization. The network architecture, dynamic anchor loss, optimizer, learning rate, batch size, learning-rate scheduler, and early-stopping settings were kept unchanged across all runs.

All ten completed runs were retained in the statistical analysis, and no run was excluded according to its test-set performance. The detailed results are summarized in Table S4. Across the ten independent runs, the normalized resonance-frequency MSE was (6.86 ± 2.67) ×10^{-5}, while the normalized spectral-waveform MSE

was (3.57 ± 0.20) ×10^{-3}. In the physical domain, the resonance-frequency RMSE was 0.0028 ± 0.0005 THz and the mean local spectral RMSE was 0.0053 ± 0.0002

These repeated-run statistics are consistent with the performance of the primary model reported in the main manuscript. In particular, the primary-model normalized resonance-frequency MSE (6.64 × 10^{-5}), normalized spectral-waveform MSE (3.58×10^{-3}), and mean local spectral RMSE (0.0054) all lie close to the corresponding ten-run central values. Therefore, the high predictive accuracy reported for Model C is not attributable to a particular random data partition or network initialization.

The repeated-training analysis serves a different purpose from the stratified bootstrap confidence intervals reported in the main manuscript. The bootstrap analysis quantifies test-sample uncertainty for the primary trained model, whereas the present ten-run experiment evaluates the reproducibility of model performance with respect to random data splitting, initialization, and training stochasticity.

Table S4. Prediction performance of Model C across ten independent training runs.

**Run**	**Normalized resonance-frequency MSE**	**Normalized spectral-waveform MSE**	**Resonance-frequency RMSE (THz)**	**Mean local spectral RMSE**
1	6.02 × 10^{-5}	3.35 × 10^{-3}	0.0027	0.0050
2	6.76 × 10^{-5}	3.80 × 10^{-3}	0.0028	0.0055
3	6.14 × 10^{-5}	3.60 × 10^{-3}	0.0027	0.0054
4	5.55 × 10^{-5}	3.19 × 10^{-3}	0.0026	0.0050
5	1.44 × 10^{-4}	3.57 × 10^{-3}	0.0041	0.0051
6	6.16 × 10^{-5}	3.80 × 10^{-3}	0.0027	0.0055
7	6.13 × 10^{-5}	3.68 × 10^{-3}	0.0027	0.0054
8	6.39 × 10^{-5}	3.72 × 10^{-3}	0.0027	0.0053
9	5.98 × 10^{-5}	3.43 × 10^{-3}	0.0027	0.0052
10	5.15 × 10^{-5}	3.57 × 10^{-3}	0.0025	0.0053
Mean ± SD	(6.86 ± 2.67) × 10^{-5}	(3.57 ± 0.20) × 10^{-3}	0.0028 ± 0.0005	0.0053 ± 0.0002
Median	6.13 × 10^{-5}	3.59 × 10^{-3}	0.0027	0.0053
Min–Max	5.15 × 10^{-5} – 1.44 × 10^{-4}	3.19 × 10^{-3} – 3.80 × 10^{-3}	0.0025 – 0.0041	0.0050 – 0.0055

Section S4. Representative spectral reconstruction by topology

Representative spectral reconstruction results were compared across the five topology classes, as shown in Figure S3. Overall, the spectra predicted by the surrogate model show excellent agreement with the resonance spectra from full-wave simulations, including peak shape, resonance frequency, and absorption amplitude. For all five topology classes, the predicted spectra closely agree with the simulated spectra, indicating that the forward surrogate model accurately reconstructs the resonance-centered local absorption response across topological structures.

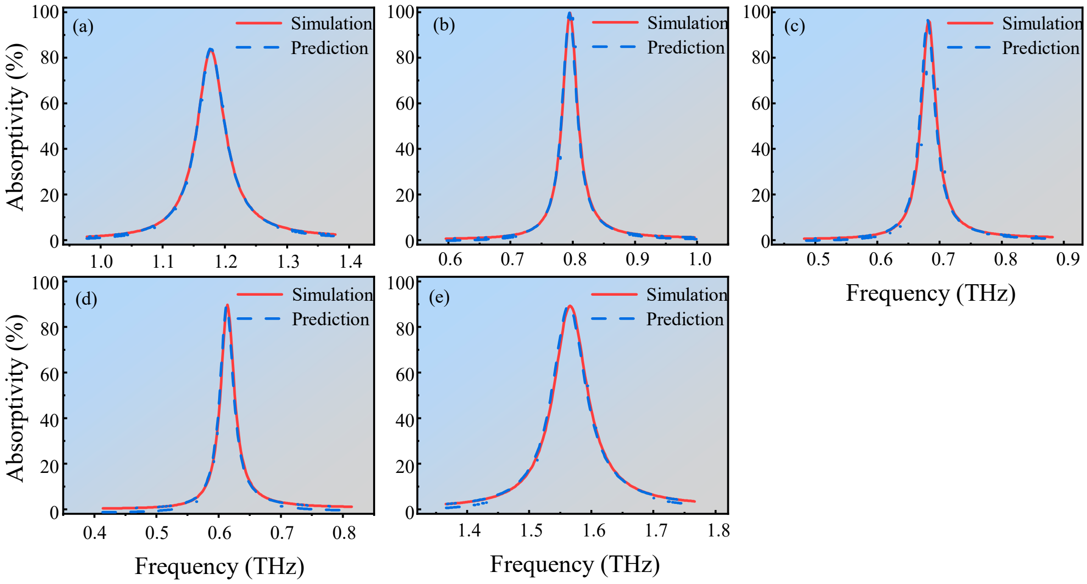

Figure S3. Representative spectral reconstruction across five topology classes. (a) Standard Cross, (b) Jerusalem Cross, (c) Cross-enclosed Split Ring, (d) Chiral Cross, and (e) Split-Cross. Red solid lines denote simulated spectra, and blue dashed lines denote predicted spectra.

Section S5. Inverse-design target selection

The objective of inverse design was not set arbitrarily; before setting the targets, a statistical examination was conducted on the resonant frequencies, peak absorption rates, and Q-values of the training dataset, along with observations of the target responses of the five topological structures, as shown in **Figure S4**. The resonance frequency range of the dataset is from 0.534 THz to 1.772 THz. As depicted in **Figure S4**(a), topology classes 4 and 5 are situated in the low- and high-frequency bands, respectively; topology 1 spans the mid-to-high-frequency range throughout the entire spectrum; and topologies 2 and 3 occupy the mid-to-low-frequency band with considerable overlap. All five topologies achieve peak absorption exceeding 99%. Notably, Structure 5 demonstrates the broadest absorption efficiency range, from 48.8% to 99.99%. The Q-factors of the structures show minimal variation, with a maximum of 29.63 and a minimum of 18.94.

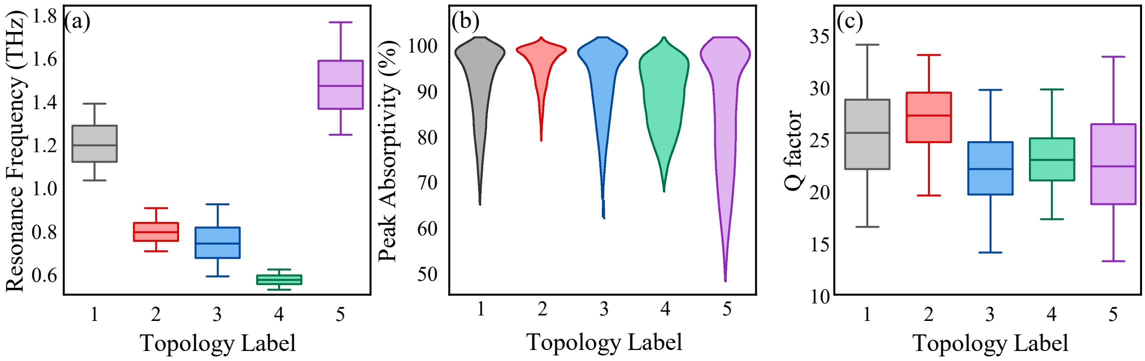

Figure S4. Training-set response distributions used for inverse-design target selection.

Section S6. Comparison with representative recent surrogate-learning and inverse-design

Recent surrogate-learning and inverse-design methods have demonstrated the potential to reduce the computational cost of metamaterial design. For example, Huang et al. combined a forward neural network with simulated annealing for terahertz absorber design [1], whereas Xing et al. used a CNN surrogate with a genetic algorithm to optimize binary metamaterial topologies [2]. These surrogate-assisted heuristic methods reduce the number of full-wave simulations and enable rapid objective evaluation, but derivative-free searches may become

inefficient in continuous design spaces and may require many objective evaluations. In addition, when the objective is defined by the global spectral error, the optimization may not sufficiently emphasize the localization of a narrow resonance.

Generative inverse-design methods can explore multiple candidate structures and accommodate highly non-unique mappings. Li et al. combined LSTM–Attention modeling, variational autoencoding, and a U-WGAN framework for the generative design of freestyle metasurfaces [3]. Ge et al. developed a self-attention conditional Wasserstein generative adversarial network for generating terahertz metamaterial sensor structures from target responses [4]. These methods can produce diverse candidate structures, but their performance depends strongly on the coverage and representativeness of the training distribution. The generated structures generally require additional forward screening and electromagnetic verification, and the response-to-structure mapping may remain sensitive to inverse non-uniqueness.

Adjoint-based optimization provides high-fidelity physical gradients and is effective for high-dimensional electromagnetic design. Jang et al. demonstrated adjoint-based inverse design for ultrathin metamaterial absorbers [5]. However, each optimization step remains coupled to full-wave or adjoint simulations, which can become computationally expensive for repeated multi-target or multi-topology searches. Jointly trained forward–inverse networks provide another route for rapidly generating candidate structures after training. Wu et al. used an MLP-based forward proxy together with a modified Swin Transformer inverse network for broadband solar metamaterial absorber design [6]. Such frameworks can impose consistency between forward and inverse mappings, but they require the simultaneous optimization and stabilization of two coupled networks. In addition, errors in the inverse network may affect the generated designs even when the forward model is accurate [6].

Compared with these approaches, the present framework introduces a coordinated design of the response representation, surrogate model, loss function, and inverse-optimization procedure. The dominant resonance frequency is predicted explicitly rather than being inferred only from the full absorption spectrum. The dynamic anchor loss further strengthens the constraints on resonance-centered spectral points, thereby reducing the risk that errors near the dominant resonance are diluted by less important spectral regions. The residual architecture improves the representation of the nonlinear parameter-to-response relationship, while the validated forward surrogate is reused as a frozen differentiable model for topology-wise projected gradient descent. This strategy avoids training a separate inverse network and avoids the derivative-free search required by simulated annealing or genetic algorithms [1-6]. At the same time, the optimized structures are independently evaluated using full-wave simulations, combining the computational efficiency of surrogate-based gradient optimization with electromagnetic verification.

The main advantage of the proposed framework is therefore not simply the use of a more complex neural network or optimizer. Rather, it lies in matching the learning and optimization strategy to the physical characteristics of narrowband resonant absorbers. By explicitly modeling the resonance frequency, emphasizing the local spectral region around the resonance, and directly optimizing the structural variables through a differentiable forward model, the framework provides a more targeted route for resonance-oriented inverse design. The resulting surrogate achieves improved resonance prediction, while the PGD-generated structures retain strong resonance behavior near the prescribed target frequencies after full-wave simulation verification.

The comparison is summarized in Table S5. Because existing studies use different materials, operating bands, structural representations, dataset sizes, spectral sampling schemes, and evaluation metrics, their reported MSE or RMSE values cannot be used to establish a strict numerical ranking. The quantitative contributions of the residual architecture and dynamic anchor loss are therefore evaluated separately through the controlled ablation study in Section 3.3 of the main manuscript.

Table S5. Methodological comparison of representative recent surrogate-learning and inverse-design approaches for metamaterial and metasurface absorbers.

Study	Design object and structural representation	Forward prediction	Response representation	Inverse design	Main methodological characteristic
Huang et al. [1]	Parameterized terahertz metasurface absorbers	Forward neural network	Absorption spectrum	Simulated annealing assisted by the forward model	Combines a neural surrogate with a heuristic optimizer for rapid forward prediction and inverse design
Li et al. [3]	Freestyle metasurface patterns	LSTM + Attention model for forward S-parameter prediction; VAE used for feature compression	S-parameter spectrum	U-WGAN-based generative inverse design	Integrates multiple neural networks to enable generative design of free-form metasurface patterns
Ge et al. [4]	Multiple terahertz metamaterial sensor structures with structural labels	No separate forward surrogate used as the primary inverse-design component	Target spectral response used as a conditional input	Self-attention conditional Wasserstein GAN	Uses label-conditioned generative learning to produce different metamaterial sensor structures from target responses
Jang et al. [5]	Ultrathin metamaterial absorbers with high-dimensional design regions	Full-wave forward and adjoint simulations rather than a learned surrogate	Absorption-related objective function	Adjoint-gradient optimization	Directly obtains physical sensitivity gradients for efficient high-dimensional electromagnetic inverse design
Wu et al. [6]	Multilayer broadband solar metamaterial absorber	MLP-based forward proxy	Broadband absorption response	Modified Swin Transformer inverse network jointly trained with the forward model	Uses consistency-driven joint forward–inverse learning to mitigate the non-uniqueness of inverse mapping
Present work	Five predefined parameterized terahertz absorber topology classes represented	Residual fully connected surrogate with a dynamic anchor loss	Resonance frequency + local absorption spectrum	Topology-wise PGD using the frozen forward surrogate	Explicit resonance-anchored response modeling and direct reuse of the validated differentiable forward surrogate for inverse optimization without training a separate inverse network

Section S7. Mesh-convergence assessment of inverse-designed absorbers

To assess whether the full-wave verification of the inverse-designed absorbers is sensitive to the numerical mesh discretization, an additional mesh-convergence analysis was performed for all five inverse-design cases T1–T5. The optimized geometries obtained from the PGD-based inverse-design procedure were retained without modification. The material properties, excitation conditions, boundary conditions, and electromagnetic solver were also kept unchanged, while the numerical mesh was progressively refined using three mesh settings, denoted as 10, 15, and 20.

For each inverse-designed absorber and each mesh setting, the absorption spectrum was recalculated using full-wave simulation. The dominant resonance frequency (f_0) was identified from the maximum absorption peak, and the corresponding peak absorptivity A_{max} was recorded. The quality factor was calculated consistently with the definition used in the main manuscript as $Q = f_0$ / FWHM, where FWHM denotes the full width at half maximum of the absorption peak.

Figure S5 compares the absorption spectra obtained using the three mesh settings for the five inverse-designed cases. In general, the resonance responses become progressively stable as the mesh is refined. For T1–T4, only small differences are observed between mesh settings 15 and 20 in the resonance frequency, peak absorptivity, and spectral linewidth. The high-frequency-edge case T5 shows a more noticeable resonance-frequency shift when the coarse mesh setting of 10 is used. Specifically, its resonance frequency changes from 1.524 THz at mesh setting 10 to 1.594 THz at mesh setting 15. However, further mesh refinement from 15 to 20 changes the resonance frequency only from 1.594 to 1.596 THz, while the overall resonance line shape, peak absorptivity, and Q-factor remain nearly unchanged. This behavior indicates that the coarse mesh is insufficient for accurately resolving the high-frequency resonance of T5, whereas the result becomes stable after further refinement.

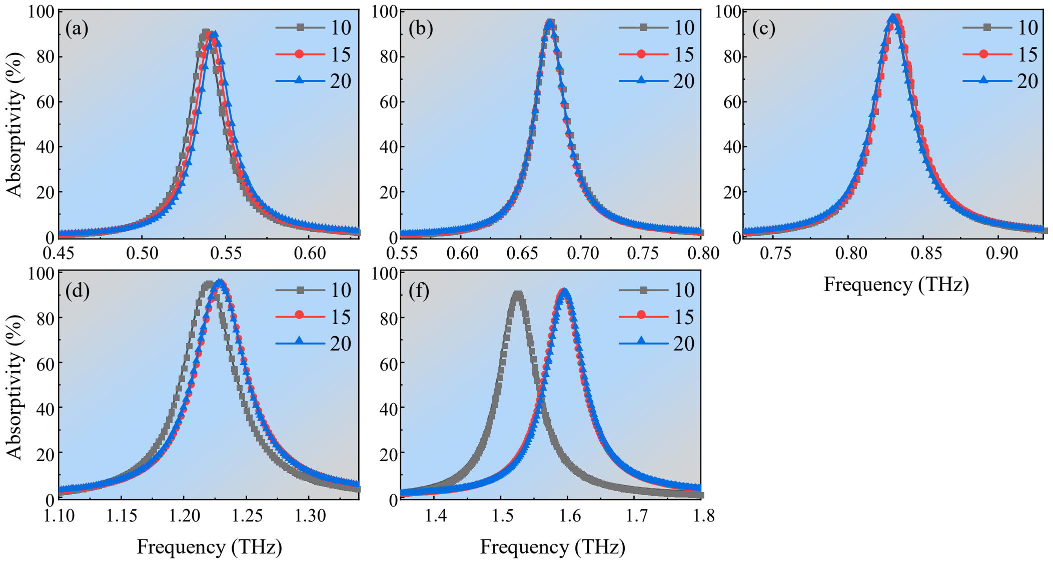

Figure S5. Mesh-convergence comparison of the absorption spectra for the five inverse-designed absorber cases. (a) T1; (b) T2; (c) T3; (d) T4; and (e) T5. For each optimized geometry, the material properties, excitation, boundary conditions, and electromagnetic solver were kept unchanged, while the numerical mesh was progressively refined using mesh settings of 10, 15, and 20. The spectra obtained using settings 15 and 20 show close agreement for all five cases, indicating numerical convergence of the principal resonance characteristics after sufficient mesh refinement. The high-frequency-edge T5 case exhibits a larger resonance-frequency shift for the coarsest mesh setting, but the response becomes stable with further refinement.

The quantitative mesh-convergence results are summarized in Table S6. When the mesh setting is increased from 15 to 20, the maximum relative variation in resonance frequency among T1–T5 is approximately 0.37%. The maximum peak-absorptivity variation is approximately 0.20 percentage points, while the maximum relative Q-factor variation is approximately 1.24%. These small changes indicate that the principal resonance characteristics of the five inverse-designed absorbers are numerically stable under further mesh refinement. Therefore, the full-wave verification of the optimized candidates is not strongly dependent on a single mesh discretization once an adequately refined mesh is employed.

Table S6. Mesh-convergence results for the five inverse-designed absorbers.

**Target case**	**Mesh setting**	f_0 **(THz)**	A_{max} **(%)**	Q
	10	0.538	91.18	22.55
T1	15	0.542	90.02	22.83
	20	0.544	89.89	22.81
	10	0.673	95.52	21.81
T2	15	0.674	95.02	22.67
	20	0.674	95.05	22.42
	10	0.829	97.63	26.43
T3	15	0.831	97.69	26.06
	20	0.830	97.56	26.38
	10	1.220	95.81	24.47
T4	15	1.230	95.40	24.80
	20	1.228	95.60	24.75
	10	1.524	91.33	22.84
T5	15	1.594	91.62	22.82
	20	1.596	91.42	22.89

Section S8. Multi-start robustness and initialization sensitivity of PGD

To evaluate the sensitivity of PGD to the initial geometry, a multi-start analysis was performed for all five inverse-design targets T1–T5. For each target, the five topology classes were independently optimized from 10 initial geometries, resulting in 50 PGD runs per target and 250 runs in total. The first restart of each topology used its training-set mean geometry, whereas the remaining nine initial geometries were uniformly sampled within the corresponding topology-specific feasible bounds. All other optimization settings were kept unchanged.

A run was considered successful when the resonance-frequency error was no greater than 0.01 THz, the absorptivity error was no greater than 0.03, and the relative Q-factor error was no greater than 10%. As summarized in Table S7, T1, T2, and T4 achieved 100% response success and 100% topology-selection consistency. T3 also achieved 100% response success, while Jerusalem Cross and Cross-Enclosed Split Ring were selected in 8 and 2 of the 10 restart rounds, respectively, reflecting the one-to-many nature of the inverse problem. T5 consistently selected the Split-Cross topology, although its combined response success rate decreased to 20%, mainly because the peak-absorptivity error was more sensitive near the high-frequency boundary.

These results indicate that the topology-wise multi-start strategy substantially reduces dependence on a single initialization for T1–T4 and maintains stable topology selection for all five targets. Since PGD is a local gradient-based optimizer, no strict guarantee of global optimality is claimed; instead, the multi-start procedure is used to identify repeatable low-loss candidates and reduce the risk of retaining a poor local solution.

Table S7. Multi-start robustness of PGD inverse design for T1–T5.

Target	Topology	Best loss	Median loss	Mean loss ± SD	Response success rate	Topology-selection consistency
T1	Chiral Cross	8.90×10^{-6}	3.25×10^{-4}	7.29×10^{-4} ± 1.46×10^{-3}	100%	100%
T2	Cross-Enclosed Split Ring	2.50×10^{-8}	4.86×10^{-4}	8.94×10^{-4} ± 1.02×10^{-3}	100%	100%
T3	Jerusalem Cross*	3.71×10^{-5}	7.81×10^{-4}	6.90×10^{-4} ± 4.78×10^{-4}	100%	80%
T4	Standard Cross	0	2.32×10^{-5}	1.72×10^{-4} ± 4.05×10^{-4}	100%	100%
T5	Split-Cross	1.07×10^{-2}	1.96×10^{-2}	1.75×10^{-2} ± 3.81×10^{-3}	20%	100%

*For T3, Jerusalem Cross was selected in 8 of the 10 multi-start rounds and Cross-Enclosed Split Ring in the remaining 2 rounds; all 10 solutions satisfied the predefined response-success criteria. A successful solution simultaneously satisfies $|f_r - f_r^*| \leq 0.01$ THz, $|A_r - A_r^*| \leq 0.03$, and $|Q_r - Q_r^*|/Q_r^* \leq 10\%$.

For T5, the resonance frequency remained stable across the multi-start solutions, with a mean error of $3.13\times10^{-4} \pm 3.61\times10^{-4}$ THz, while the mean relative Q-factor error was 4.18% ± 0.59%. The reduced success rate was mainly caused by the peak-absorptivity error (0.0298 ± 0.0030), which lies close to the prescribed tolerance and is consistent with the increased difficulty of the high-frequency boundary case.

References

1. Huang, Y.; Liu, X.; Liu, M.; Chen, J.; Du, W.; Liu, Z. Deep learning-based inverse design of multi-functional metasurface absorbers. Optics Letters 2024, 49, 2733, doi:10.1364/ol.518786.
2. Xing, R.; Zou, X.; Fei, Y.; Zhang, Y.; Chen, L. Functional design of metamaterial absorbers using a combined convolutional neural network-genetic algorithm approach. Optics Express 2026, 34, 18384-18395.
3. Li, J.; Yang, C.; Qinhua, A.; Lan, Q.; Yun, L.; Xia, Y. On-Demand Design of Metasurfaces through Multineural Network Fusion. ACS Applied Materials & Interfaces 2024, 16, 49673-49686, doi:10.1021/acsami.4c11972.
4. Ge, H.; Bu, Y.; Ji, X.; Jiang, Y.; Jia, K.; Zhang, Y.; Zhang, Y.; Wu, X.; Sun, Q. Inverse Design of Multistructured Terahertz Metamaterial Sensors Based on Improved Conditional Generative Network. ACS Applied Materials & Interfaces 2024, 16, 60772-60782, doi:10.1021/acsami.4c10921.
5. Jang, E.; Cho, J.; Kang, C.; Chung, H. Inverse Design of Ultrathin Metamaterial Absorber. Nanomaterials 2025, 15, 1024.
6. Wu, Q.; Deng, Z.; Zeng, C.; Cai, H. Accurate Inverse Design of Broadband Solar Metamaterial Absorbers via Joint Forward–Inverse Deep Learning. Nanomaterials 2026, 16, 297.
